# Super enhancer-driven core transcriptional regulatory circuitry crosstalk with cancer plasticity and patient mortality in triple-negative breast cancer

**DOI:** 10.3389/fgene.2023.1258862

**Published:** 2023-10-12

**Authors:** Wensheng Shi, Bowen Zhong, Jiaming Dong, Xiheng Hu, Lingfang Li

**Affiliations:** ^1^ Department of Dermatology, Hunan Engineering Research Center of Skin Health and Disease, Hunan Key Laboratory of Skin Cancer and Psoriasis, Xiangya Hospital, Central South University, Changsha, Hunan, China; ^2^ National Engineering Research Center of Personalized Diagnostic and Therapeutic Technology, Central South University, Changsha, Hunan, China; ^3^ Furong Laboratory, Changsha, Hunan, China; ^4^ Department of Urology, Xiangya Hospital, Central South University, Changsha, Hunan, China; ^5^ Department of Radiation, Cangzhou Central Hospital, Changsha, China; ^6^ Department of Cardiovascular Medicine, Xiangya Hospital, Central South University, Changsha, China

**Keywords:** cancer plasticity, triple-negative breast cancer, patient mortality, core transcriptional regulatory circuitry, super enhancer

## Abstract

Triple-negative breast cancer (TNBC) is a clinically aggressive subtype of breast cancer. Core transcriptional regulatory circuitry (CRC) consists of autoregulated transcription factors (TFs) and their enhancers, which dominate gene expression programs and control cell fate. However, there is limited knowledge of CRC in TNBC. Herein, we systemically characterized the activated super-enhancers (SEs) and interrogated 14 CRCs in breast cancer. We found that CRCs could be broadly involved in DNA conformation change, metabolism process, and signaling response affecting the gene expression reprogramming. Furthermore, these CRC TFs are capable of coordinating with partner TFs bridging the enhancer-promoter loops. Notably, the CRC TF and partner pairs show remarkable specificity for molecular subtypes of breast cancer, especially in TNBC. USF1, SOX4, and MYBL2 were identified as the TNBC-specific CRC TFs. We further demonstrated that USF1 was a TNBC immunophenotype-related TF. Our findings that the rewiring of enhancer-driven CRCs was related to cancer immune and mortality, will facilitate the development of epigenetic anti-cancer treatment strategies.

## Introduction

Triple-negative breast cancer (TNBC), an aggressive subtype of breast tumor that lacks hormone receptor expression and HER2 gene amplification, accounts for 12%–18% of breast neoplasms (Foulkes et al., 2010). Multiomics contributed to the development of precision medicine by providing new insights into the biology and heterogeneity of TNBC. Transcription factors (TFs) play a vital role as key constituents within regulatory gene transcription networks (Lambert et al., 2018). The regulatory complexes that TFs frequently create with proteins bind the promoters or enhancers of genes, take part in gene transcription, and have an impact on gene expression (Feng et al., 2023). Core transcriptional regulatory circuitry (CRC), which consists of the core TFs, is an interconnected autoregulatory loop in which the TFs self-regulate by binding to their own super-enhancers (SEs) and the TFs themselves bind to the SEs of one another (Li et al., 2021). Understanding the regulatory mechanisms and functional implications of SEs and CRC TFs in breast cancer subtypes is essential for unraveling the complexity of this disease and identifying potential therapeutic targets.

In this study, we investigated the role of SEs and CRC TFs in breast cancer, with a specific focus on the molecular subtypes and their associated clinical characteristics. These CRC TFs established self-regulatory loops and collaborated with other TFs to govern the communication between enhancers and promoters, enabling reciprocal regulation and involvement in the reprogramming of gene expression in cancer. TNBC exhibited unique CRC TF profiles compared to other subtypes and its specific CRC TFs participated in various regulation of gene expression in different biological processes. Understanding the molecular mechanisms underlying these regulatory networks can contribute to the development of targeted therapies and improved clinical management strategies for breast cancer patients, especially those with TNBC.

## Material and methods

### Data acquisition and preprocessing

The Cancer Genome Atlas (TCGA) level 3 gene expression, DNA methylation, and the copy number variation (CNV) data of Breast invasive carcinoma (BRCA) were obtained from the UCSC Xena browser (http://xena.ucsc.edu). The enhancer RNA (eRNA) transcription data of the corresponding TCGA samples were downloaded from the Cancer eRNA Atlas (TCeA) (Chen and Liang, 2020). The ChIP-seq bigWig files of DNase (ENCSR000EPH), EP300 (ENCSR000BTR), H3K27ac (ENCSR752UOD), H3K4me1 (ENCSR493NBY), H3K4me2 (ENCSR875KOJ), and H3K4me3 (ENCSR985MIB) in MCF-7 cells were downloaded from ENCODE (Davis et al., 2018). For the gene expression profiles, we required the fraction of gene expression (FPKM > 0) over 70%.

### Identification of activated super-enhancers

**FIGURE 1:**
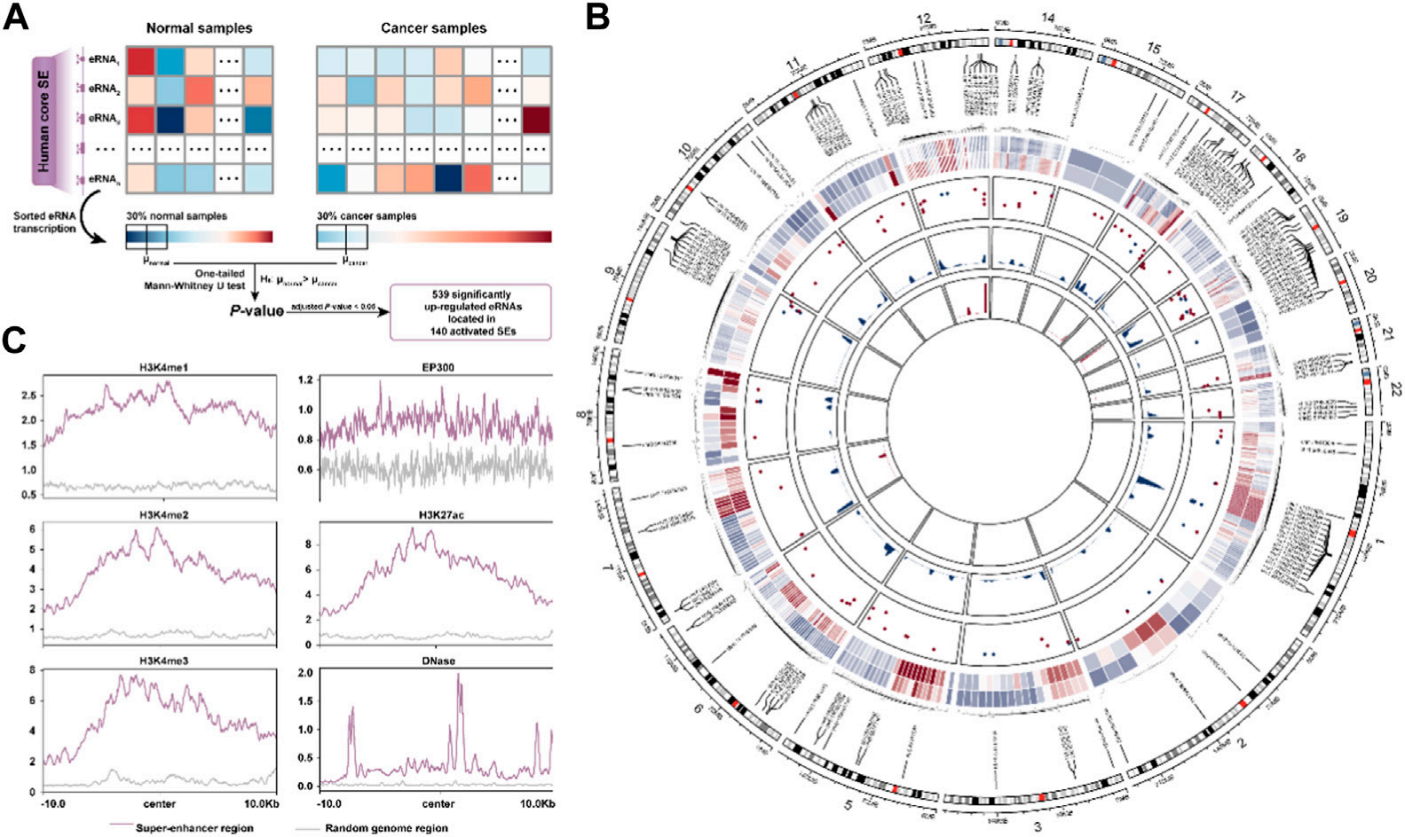
The activated super-enhancer chromatin landscape in BRCA. **(A)**. Schematic diagram for identification of activated SE in BRCA. **(B)**. The chromatin landscape of activated SE. Circos plot’s tracks are, from outer to inner, the average transcript levels of eRNA in normal and cancer samples, the frequency of CNV amplification (red) and deletion (blue) in the SE region, the density plot of delta β-value for hypomethylated CpG sites, and the hypermethylated CpG sites, respectively. **(C)**. Distribution of activity-associated modification marks around SE regions.

The human core super-enhancer (SE, n = 1,531) list was obtained from the recent study of Chen and Liang (2020). There were 7,379 eRNA loci were mapped in the 1,257 core SE genome regions. The eRNAs that were not transcribed (RPKM = 0) in > 70% samples were removed. To identify the activated SEs in cancer samples, we tested whether the mean eRNA transcription level in 30 % of cancer samples with the lowest transcription levels ( $\mu_{c}$ ) was higher than that in 30% normal samples with the lowest transcription levels ( $\mu_{n}$ ) using the unpaired one-tailed Mann-Whitney U test. We used 30 % as a cut-off, which would allow us to immune against the effect of prevalently low transcription levels of eRNA, while also yielding sufficient statistical power (Yao et al., 2015). The *p*-values were corrected using the Benjamini and Hochberg (BH) adjustment (Figure 1A). The eRNAs with adjusted *p-*values < 0.05 were considered as the significantly up-regulated eRNAs, and the SEs where these eRNAs are located were defined as the activated SEs in BRCA.

### Copy number variation analysis

We first distinguished the copy number variation (CNV) segments (amplification and deletion) based on the log2 ratio cut-off of +/− 0.8 (Bambury et al., 2015). BEDtools was used to assign the activated SE and random genome regions to CNVs-associated segments (Quinlan and Hall, 2010). Furthermore, the enrichment analysis of amplification/deletion segments between SE and random genome regions was perform based on Fisher’s exact test.

### Differential methylation analysis

We mapped the genomic coordinates of CpG sites in the Illumina Infinium HumanMethylation450 BeadChip (NCBI/GEO record GPL13534; human genome release hg19/GRCh37) to the activated SE regions. For each CpG site within the activated SE regions, we tested whether the mean β-value in 30 % of cancer samples with the lowest methylation levels ( $\mu_{c}^{\prime }$ ) was lower/higher than that in 30 % of normal samples with the lowest methylation levels ( $\mu_{n}^{\prime }$ ), respectively, based on the unpaired one-tailed Mann-Whitney U test (Yao et al., 2015). The *p*-values were corrected using the BH adjustment. The CpG sites with $\mu_{c}^{\prime }$ significantly lower than $\mu_{n}^{\prime }$ (adjusted *p-*values < 0.05) were labeled as the differentially hypomethylated CpG sites and conversely as differentially hypermethylated CpG sites.

### Recognition of SE-assigned genes

We first used the 3D genomic data of breast cancer cell lines (ChIA-PET: ENCSR200VHL, ENCSR059HDE, ENCSR403ZYJ, and ENCSR499JGQ; Hi-C: ENCSR549MGQ) to confirm the spatial proximity between SEs and promoter regions (transcription start site [TSS] ± 3 kb) of candidate SE-assigned genes. Next, we calculated the mutual information (MI) between the expression of SE component eRNAs and candidate SE-assigned genes. MI could reflect the correlation strength, i.e.,

$$I\left(SE,G\right)=\sum_{i=1}\sum_{j=1}p\left({se}_{i},g_{j}\right)\frac{p\left({se}_{i},g_{j}\right)}{p\left({se}_{i}\right)*p\left(g_{j}\right)},$$ (1)

where *se* denotes the ith eRNA transcription in an SE, g indicates jth candidate gene expression and $p\left(se,g\right)$ is the joint probability between the events. To further evaluate the significance of the MI, we applied the random permutations and Fisher’s Z statistics to calculate the *p*-value (Zhang et al., 2012). Specifically, we randomly shuffled the vectors *SE* and *G* 1,000 times and computed the random MI ( $I_{r}$ ) and the observed MI value between the observed *SE* and *G* value was transformed it into Z-value. The *p-*value was calculated as follows:

$$P=2*\varphi \left(-\left|\frac{Z-z^{\prime }}{\sigma_{z}}\right|\right),$$ (2)

where $z^{\prime }=\ln\left(1+I_{r}\right)-\ln\left(1-I_{r}\right)/2$ , $\sigma_{z}$ is the standard deviation of z, φ represents the normal cumulative distribution function. The MI analysis was performed using the “mi” function in R package MICMIC (Zhang et al., 2012). Furthermore, we identified the genes with significantly enhanced expression by SE assigned by integrating an enhanced score (eS), i.e.,

$$eS=\frac{MI*\log_{2}FC}{\varphi \left(P\right)*\varphi \left(q\right)},$$ (3)

where *P* denotes the significance levels of MI (*p* < 0.0001), *q* represents the *p*-value of the one-tailed Mann-Whitney U test corrected for BH adjustment (*q* < 0.0001, H_0_: $\mu_{c}\leq \mu_{n}$ ), φ indicates the normal cumulative distribution function (“pnorm” function in R software), and *FC* indicates the fold change of gene expression ( $FC=\mu_{c}/\mu_{n}$ ). We defined an eS threshold to reflect the gene enhanced strength by SEs as $\frac{mean\left(MI\right)*mean\left(\log_{2}FC\right)}{pnorm\left(0.0001\right)*pnorm\left(0.0001\right)}=0.005$ . The genes with eS > 0.005 were considered as the SE-assigned genes.

### Identification of core transcriptional regulatory circuitry

We first identified the master TFs from the SE-assigned TF genes. The list of human transcription factors (TFs) was obtained from AnimalTFDB (Zhang et al., 2012). Then, we calculated the expression ratio of each TF, which was defined as the fraction of cancer samples with the TF expression level passes a TF-dependent threshold. The threshold was determined as the larger value between 1 FPKM and $\mu_{c}$ of each TF (Mei et al., 2017). Finally, we evaluated the master score (MS) of TF, i.e.,

MS=λ*expression ratio, (4)

where $\lambda =\mu_{c}*\log_{2}FC$ . The MS score reflects whether master TF is commonly highly expressed in cancer. The higher the expression level, the higher the fraction of highly expressed samples, and the more significant fold change of TF expression, the greater the MS score. We defined the master TF as the TF with the top 15% MS score.

We used the FIMO software (Bailey et al., 2009) with default settings to identify the master TF binding motifs annotated by the JASPAR (Fornes et al., 2020) and Factorbook (Wang et al., 2013) in the SE regions. Genomic coordinates of GRCh37 were transformed to GRCh38 using the UCSC liftOver tool (Haeussler et al., 2019). The genome sequences of the SE regions were extracted from the GENCODE GRCh38 reference genome sequence using Samtools (Li et al., 2009; Harrow et al., 2012). To reduce random background noise, we only considered the valid motifs that occurred at least 5 times and the FIMO outputs with *p* < 0.0001. Finally, we defined the core transcriptional regulatory circuitry (CRC) as the master TFs, which are capable of binding to SEs to co-regulate their own gene expression, thus forming an interconnected autoregulatory loop.

### Dissection of downstream genes perturbed by CRCs

We scanned the motifs of CRC TFs in SE and promoter regions of differentially expressed genes (*q*-value < 0.01, Mann-Whitney U test) as described above to obtain the candidate downstream genes perturbed by CRCs. Next, we calculated the MI between the expression levels of CRC TFs and downstream genes. The *p*-value was adjusted by 1,000 random permutations. The gene with adjusted *p*-value < 0.01 was considered as the downstream genes perturbed by CRCs. Furthermore, we annotated the function classifications of downstream genes in CR2Cancer (Ru et al., 2018) (Chromatin Modulators), KEGG (Kanehisa et al., 2004) (Signaling pathway and Metabolism), and AnimalTFDB (Zhang et al., 2012) (TFs and TF cofactors).

### Identification of CRC TF-partner TF pair bridging the SE-promoter loop

We first identified the partner TFs with 14 CRC TFs according to the following conditions: (1) The partner TFs could interact with CRC TFs by integrating the experimentally detected protein-to-protein physical interactions (PPIs) from the Human Reference Interactome (HuRI) map (Luck et al., 2020), the Biological General Repository for Interaction Datasets (BioGRID) (Oughtred et al., 2019), and the APID Interactomes (Alonso-López et al., 2019). PPIs appearing in at least two datasets were retained. (2) The expression ratio of partner TFs should over 0.3 in cancer samples. (3) The motifs of partner TFs represented at least 5 times in SE or promoter regions of SE-assigned genes using the FIMO software (Bailey et al., 2009). (4) There were significantly expressed associations between partner TFs and their corresponding SE-assigned genes by calculating the MI (*p* < 0.01).

Subsequently, we determined the CRC TF-partner TF pairs (CTPs) which contribute to bridging the SE-promoter loops by employing the partial rank correlation analysis. In brief, the CRC TFs and their interacted partner TFs could bind to the SE and its assigned genes’ promoters, respectively. The transcription levels of eRNA *i* located in the SE regions and corresponding SE-assigned gene *j* across cancer samples were defined as *SE*(*i*) and *G*(*j*), separately. The expression levels of CTPs were defined as TF(*n*). The partial correlation coefficient ( $\rho \left(SE,G|TF\right)$ ) was calculated between the transcription levels of eRNA *i* and corresponding SE-assigned gene *j* by removing the effect of the TF pairs *n*, i.e.,

$$\rho \left(SE,G|TF\right)=\frac{\rho \left(SE,G\right)-\rho \left(SE,TF\right)\rho \left(G,TF\right)}{\sqrt{\left(1-\rho_{\left(SE,TF\right)}^{2}\right)\left(1-\rho_{\left(G,TF\right)}^{2}\right)}},$$ (5)

where $\rho \left(SE,G\right)$ , $\rho \left(SE,TF\right)$ , and $\rho \left(G,TF\right)$ represent the Spearman’s correlation coefficient between the eRNA and SE-assigned gene, between the eRNA and TF, and between the SE-assigned gene and TF. In addition, we obtained the *p*-value for the $\rho \left(SE,G\right)$ as $P_{r}$ and $\rho \left(SE,G|TF\right)$ as $P_{p}$ , respectively. The CTPs with $P_{r}<0.05$ and $P_{p}>0.05$ were considered as the key factors of bridging SE-promoter loops. Besides, we applied the conditional independence test to validate the dependence relationships of SE and SE-assigned genes on CTPs (Tsamardinos and Borboudakis, 1970). More than 67.3% of dependence relationships could be confirmed by the conditional independence test.

### Functional enrichment analysis

The functional enrichment analysis was performed by Metascape webserver (Zhou et al., 2019). We enriched each given gene list to the following ontology sources: KEGG Pathway, GO Biological Processes, Reactome Gene Sets, Canonical Pathways, and CORUM. The accumulative hypergeometric distribution was used to calculate the enrichment significance (*p*-values). Terms with *p*-value < 0.01, minimum count of 3 were considered as the significantly enriched processes.

## Results

### Activated super-enhancer shows correlation with DNA hypomethylation and copy number amplification in BRCA

We first interrogated the activated super-enhancers (SEs) in BRCA from the 1,531 human core SEs defined by Chen and Liang (2020) based on their eRNA transcription levels (Figure 1A, See the “Methods” section). A total of 140 activated SEs were identified covering 539 significantly up-regulated eRNAs. To better understand the abnormal activation of SE, we portrayed the activated super-enhancer chromatin state landscape from a multi-omics perspective. We found that the overall copy number level of SE was significantly higher than that of random genomic regions (*p* < 2.2e-16, Mann-Whitney U test) (Supplementary Figure S1A). The CNV amplification segments were more significantly enriched to these activated SE regions (OR = 27.1, *p* < 2.2e-16, Fisher’s exact test). There was also a positive correlation between the eRNA transcription and CNV amplification (Supplementary Figure S1B). Moreover, we investigated the DNA methylation levels in SE regions. 322 CpG sites were mapped to our SE regions including 196 hypomethylated and 12 hypermethylated CpG sites. The methylation level of SE was significantly lower than that of random genomic regions (*p* = 0.001) (Supplementary Figure S1C, Figure 1B). The overall methylation levels of these sites in cancer samples were also lower than those in normal samples (Supplementary Figure S1D). Finally, the ChIP-seq data showed the SE regions were enriched with higher active signals (H3K4me1, H3K4me2, H3K27ac, and EP300), transcriptional signals (H3K4me3), along with enhanced chromatin accessibility (DNase) compared to the random genomic region (Figure 1C). These results suggested the activated SEs are closely associated with the CNV amplification, DNA hypomethylation, and various active chromatin modification signals.

### Super-enhancer regulates the expression of breast cancer-associated gene

**FIGURE 2:**
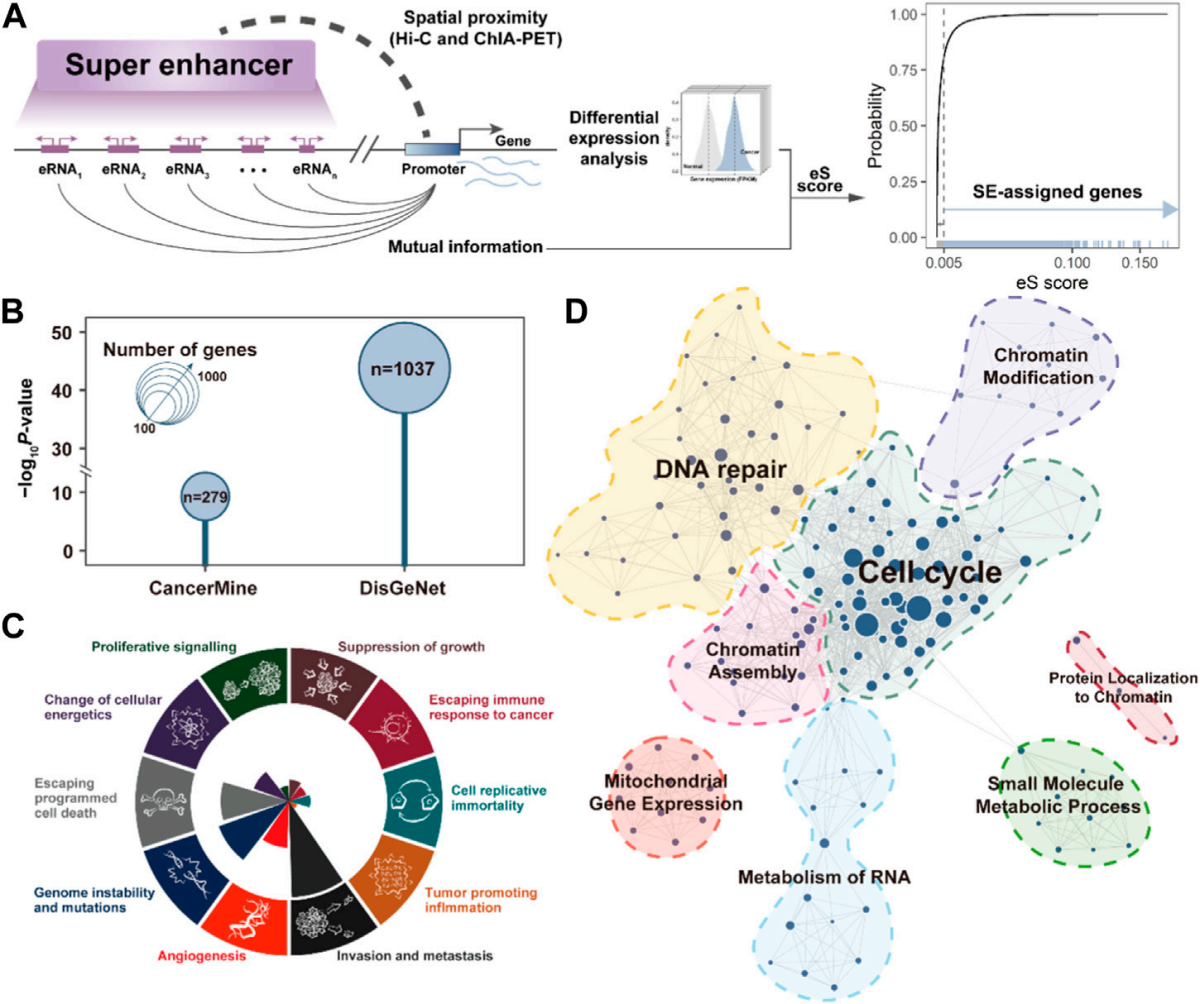
SE-assigned genes are associated with cancer processes. **(A)**. The pipeline for identifying the SE-assigned genes. **(B)**. Bubble plot showing the enrichment results between SE-assigned genes and CancerMine/DisGeNet breast cancer-related genes. *p*-value is calculated by the Fisher exact test. **(C)**. The number of SE-assigned genes that contribute to the significantly-enriched cancer hallmarks. **(D)**. SE-assigned gene-related functional module networks.

To better understand the biological functions of SEs in cancer development, it is essential to identify genes that are perturbed by SEs. Based on the 3D genomic and TCGA transcriptome data, we required the SEs should be spatially proximate to their assigned target gene promoters, and there was a significant association between the expression of SE component eRNA and SE-assigned gene by calculating their mutual information (Figure 2A). A total of 2,763 SE-assigned genes were identified as aberrantly upregulated in BRCA. Notably, these SE-assigned genes significantly enriched in breast cancer-related cancer genes recorded in CancerMine (*p* = 5.14e-10) (Lever et al., 2019) and DisGeNet (*p* = 1.52e-44, Fisher’s exact test) (Pinero et al., 2020), implying the SEs play an important role in cancer development (Figure 2B). Besides, we analyzed the cancer hallmarks of Cancer Gene Census (Santarius et al., 2010) in the SE-assigned genes, which mainly present in the “Genome instability and mutations”, “Escaping programmed cell death”, and “Invasion and metastasis” (Figure 2C). Finally, we also further annotated the potential biological functions involved in SEs and clustered similar terms to construct the SE-related functional modules in BRCA, i.e., Cell Cycle, DNA repair, Chromatin Modification, etc (Figure 2D). These results suggested the activated SE are broadly involved in multiple cancer-related processes, and plays a critical role in breast cancer development.

### Super-enhancers drive core transcriptional regulatory circuitry contributing to cancer reprogramming

**FIGURE 3:**
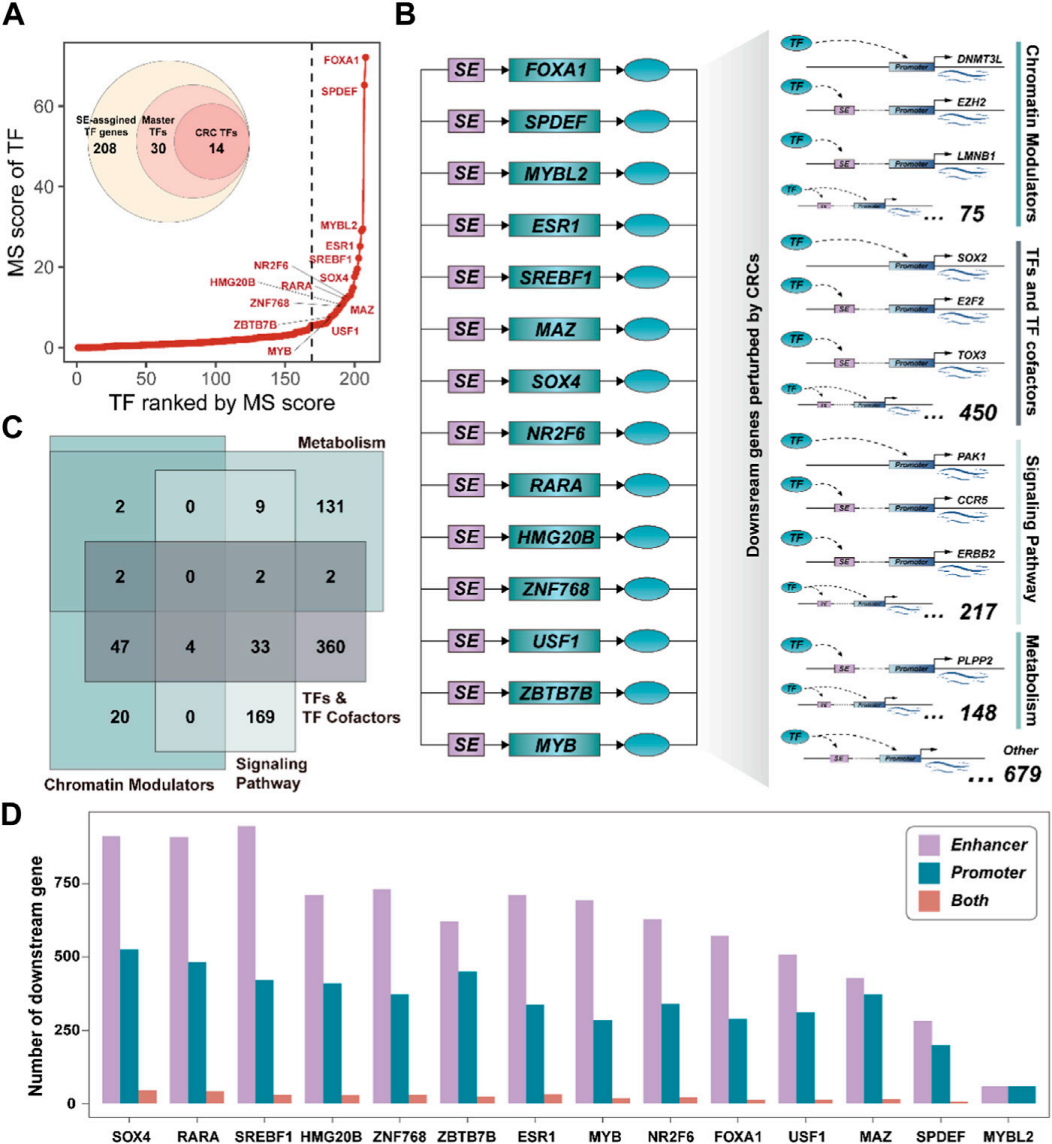
Core transcriptional regulatory circuits could be involved in cancer reprogramming. **(A)**. The ranked master TF plot by the MS score. The CRC TFs are marked with their gene symbol. **(B)**. Schematic diagram of the CRC and the downstream target genes it perturbs. **(C)**. Venn plot showing the classifications of downstream genes perturbed by CRCs. **(D)**. Bar plot showing the number of downstream genes targeted by each CRC TF by binding to enhancer or promoter.

Among all SE-assigned genes, 208 TF genes (∼8%) were identified, which may play a critical regulator in the development of BRCA. We, thus, systemically interrogated the master TFs by calculating the Master score (MS) (See the ‘Methods’ section). A total of 30 SE-assigned TF genes were considered as the master TFs, including some core TFs have been demonstrated by previous studies, i.e., FOXA1 (Arruabarrena-Aristorena et al., 2020), ESR1 (Robinson et al., 2013), and MYB (Cic et al., 2021), etc. (Figure 3A). Notably, 14 master TFs could feedback bind to their corresponding SEs, forming positive autoregulatory loops (See the ‘Methods’ section). These self-regulating master TFs were also capable of regulating each other (Supplementary Figure S2). The interconnected autoregulatory loop had been defined as the core transcriptional regulatory circuitry (CRC) by previous studies (Saint-André et al., 2016) (Figure 3B). Subsequently, we determined the downstream genes perturbed by CRCs by dissecting the binding motif of each CRC TF in promoter and SE regions (See the “Methods” section). There were 3,405 downstream genes could be perturbed by CRCs, including 2,325 bound to SEs, 947 bound to promoters, and 133 bound to both SEs and promoters (Supplementary Figure S3A), suggesting CRC TF was more likely to influence gene expression programs by binding to SE regions.

Besides, we annotated the biological functions of downstream genes for each CRC TF. These downstream genes were closely associated with DNA conformation change, metabolism processes, and signaling response, etc. (Supplementary Figure S3B), implying they could be mainly attributed to Chromatin Modulators, TFs, and TF cofactors, Metabolism, and Signaling pathway. Notably, we also observed most of them could act as TF and TF cofactors, which further highlighted that CRC could act as the critical driver of gene expression programs by regulating the expression of other downstream regulator genes (Figure 3C). Furthermore, there were 21 regulatory relationships among these CRC TFs. Remarkably, CRC TF SOX4 could act as the downstream genes of multiple CRC TFs (Supplementary Figure S3C) and it could regulate the largest number of downstream genes compared to other CRC TFs (Figure 3D). Previous studies have shown that SOX4 is an essential developmental transcription factor and is frequently overexpressed as an oncogene in more than 20 malignancies (Moreno, 2020). These results further suggested that CRC TFs can inter-regulate and participate in gene expression reprogramming in cancer.

### CRC TFs coordinate with partner TFs participating in enhancer-promoter communications

**FIGURE 4:**
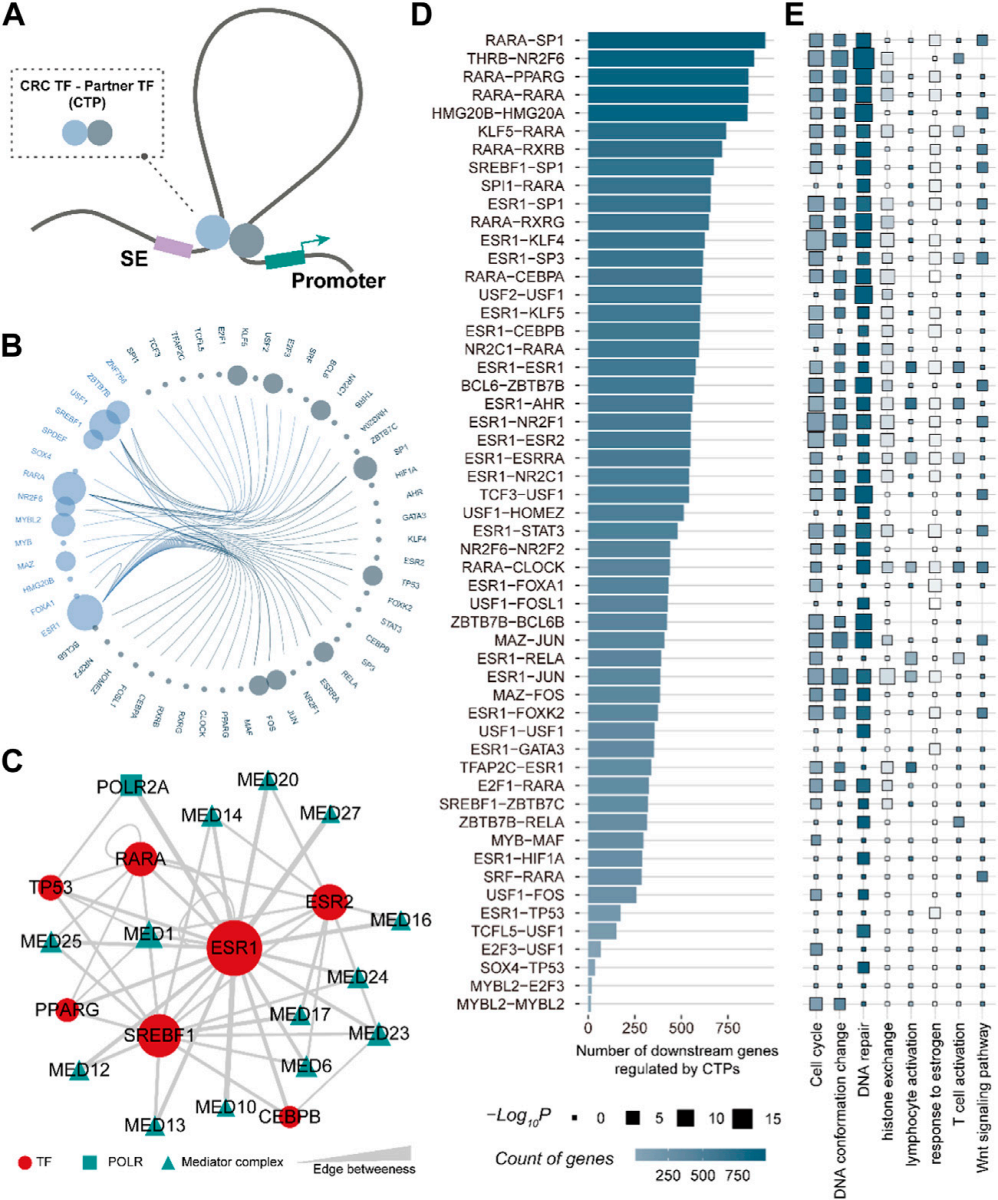
CRC TF is capable of bridging the enhancer-promoter communications. **(A)**. The 3D genome model of the CRC TF communicating enhancer-promoter loop. **(B)**. Edge bundling showing the CRC TF-partner TF pairs. The node size denotes the number of CTPs constructed by the TF. **(C)**. CRC TFs ESR1-, RARA-, and SREBF1- related CTP could be linked by the Mediator complex. **(D)**. Bar plot showing the number of downstream genes regulated by CTPs. **(E)**. The GO biological processes are enriched by downstream genes of each CTPs.

TFs are defined by their ability to bind to DNA but typically function through interactions with other proteins (Lambert et al., 2018). To better characterize the role of CRC TF in enhancer-promoter communications, we systemically interrogated the partner TFs for each CRC TF by employing the 3D genome model and partial correlation analysis (Figure 4A) (See the “Methods” section). A total of 52 TFs were identified as CRC TF partners. They could interact with CRC TFs via direct protein-to-protein interactions, forming 54 CRC TF-partner TF pairs (CTPs) involved in bridging the enhancer-promoter loops (Figure 4B). Notably, ESR1 had the greatest number of partners, comprising 20 CTPs, of which the ESR1-GATA3 pair had been demonstrated that could regulate gene expression by shaping enhancer accessibility in breast cancer (Theodorou et al., 2013). Besides, 2,181 SE-assigned genes (∼80%) could be regulated by CTPs. Each gene was modulated by an average of 11.9 CTPs, suggesting that there was also general cooperation between CTPs. We further analyzed the linkages between CTPs by considering the RNA polymerase (POLR2A) and the Mediator complex as mediators. The Mediator complex was extensively involved in the linkages between CRC TFs ESR1-, RARA-, SREBF1- related CTPs, which consistent with the previous studies (Kagey et al., 2010) (Figure 4C). Moreover, the CTP RARA-SP1 had the largest number of downstream genes across all the CTPs (Figure4D). The downstream genes regulated by CTPs are also enriched in cell cycle, DNA conformation change, DNA repair, etc. (Figure 4E). These results showed that the CRC TFs could widely coordinate with other TFs bridging the enhancer-promoter communications participating in multiple cancer processes.

### Triple-negative breast cancer shows distinctive CRC TFs compared to other BRCA molecular subtypes

**FIGURE 5:**
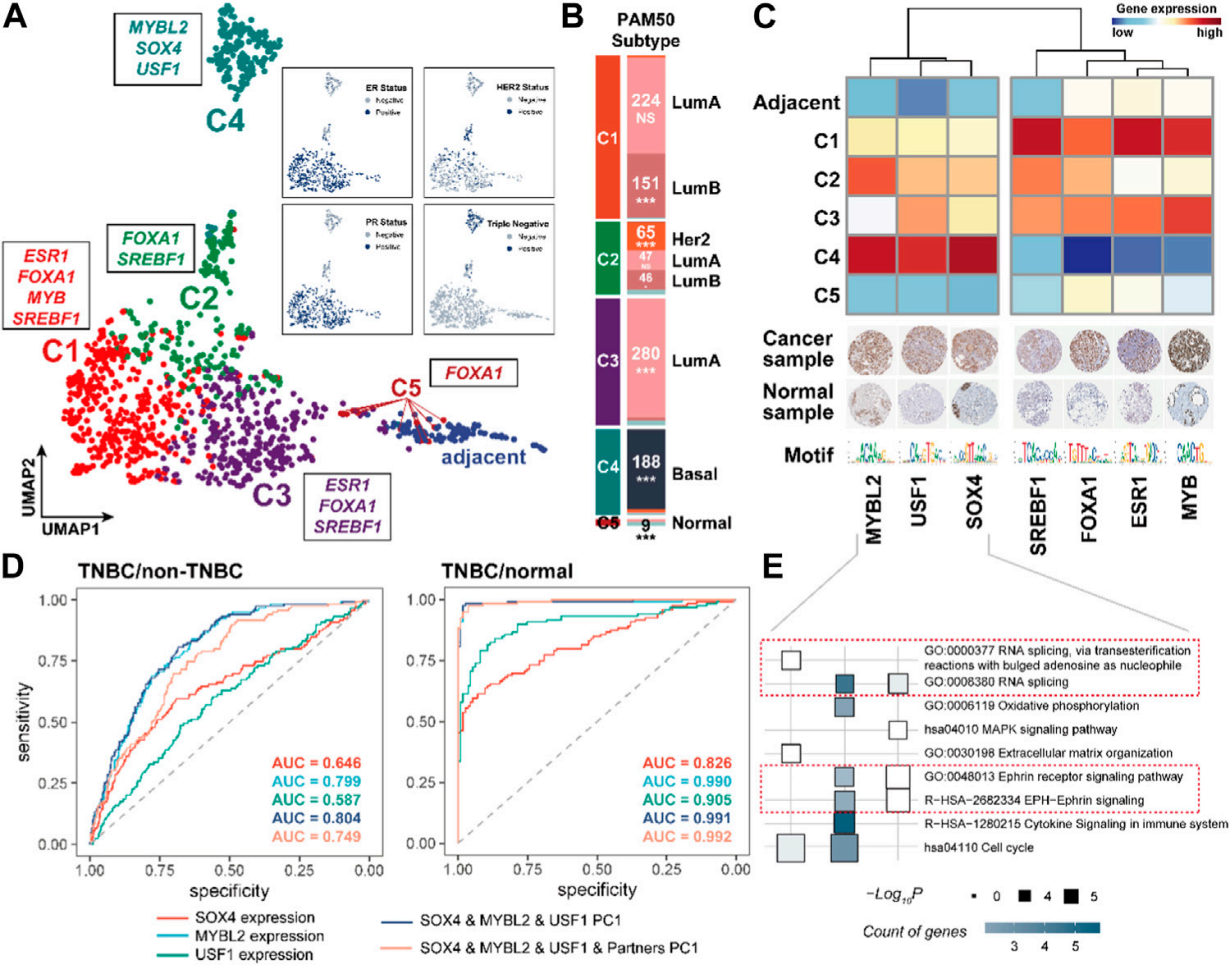
CRC TFs could contribute to the molecular and PAM50 subtypes of BRCA. **(A)**. Consensus and two-dimensional embedding clustering (UMAP) grouped BRCA samples into five robust CRC subtypes. **(B)**. Enrichment between the CRC subtypes and PAM50 subtypes. ^*^ *p* < 0.05, ^**^ *p* < 0.001, and ^***^ *p* < 0.0001, as calculated by accumulative hypergeometric distribution. **(C)**. Heatmap showing the subtype-specific CRC TFs expression levels. The IHC staining of each CRC TF protein in both breast cancer samples and normal tissues was shown below. For full IHC protein profiles, view the gene at www.proteinatlas.org/pathology. **(D)**. ROC curves for identifying the TNBC and non-TNBC samples, and TNBC and normal samples based on the SOX4, MYBL2, USF1 expression, PC1 of SOX4, MYBL2, and USF1, PC1 of SOX4, MYBL2, USF1, and their partners, respectively. **(E)**. Functional analysis of downstream genes of each CRC.

We next performed the consensus clustering analysis on 1,104 BRCA samples using the expression of CRC TFs and their partner TFs (Supplementary Figures S4A–C). The analysis and two-dimensional embedding clustering grouped samples into five robust CRC subtypes (C1, C2, C3, C4, and C5) (Figure 5A). Notably, the CRC subtypes had obvious overlap with the known molecular subtypes of breast cancer defined by the expression of estrogen receptor (ER) or progesterone receptor (PR) and human epidermal growth factor receptor 2 (Her2) including ER/PR+, Her2+; ER/PR+, Her2−; ER/PR−, Her2+; and ER/PR−, Her2−. The samples in C1, C3, and C5 were closely associated with the ER/PR+, Her2− subtype; those in C2 were related to the Her2+ subtype; and those in C4 mainly showed the ER/PR−, Her2− subtype (triple-negative). Besides, the CRC subtypes were significantly enriched in the PAM50 subtypes {Luminal A [LumA], Luminal B [LumB], Her2-enriched [Her2+], Basal-like, and Normal-like (Perou et al., 2000; Sørlie et al., 2001)} based on the accumulative hypergeometric distribution. We found that C1 was significantly enriched to “LumB”; C2 was enriched to “Her2+” and “LumB”; C3 was enriched to “LumA”; C4 was enriched to “Basal-like”, and C5 was enriched to “Normal-like” (Figure 5B). Previous studies have shown that 71% of triple-negative breast cancer (TNBC) were found to be “Basal-like” while 77% of “Basal-like” cancers were triple-negative (Bertucci et al., 2008). And C4 is clearly distinguished from other subtypes in two dimensions (Figure 5A). Hence, we considered that C4 was a TNBC-enriched type. These indicate that CRC TFs and their partners have good performance for distinguishing the clinical subtypes of BRCA.

We subsequently recognized the subtype-specific CRC TF modules, i.e., C1: FOXA1, SREBF1, ESR1, and MYB; C2: FOXA1, SREBF1; C3: FOXA1, SREBF1, and ESR1; C4: SOX4, USF1, and MYBL2; and C5: FOXA1 (Supplementary Figure S5A). Remarkably, the C4 subtype-specific CRC TFs were obviously distinguished from other subtypes, implying the distinct gene expression program of TNBC (Figure 5C). We also observed the C4 subtype-specific CRC TFs and their partner TFs were significantly dysregulated compared to non-TNBC and normal samples, in which SOX4, USF1, and MYBL2 were significantly upregulated in TNBC samples (Supplementary Figure S5B). Besides, the first principal component (PC1) based on the expression of SOX4, USF1, and MYBL2 was a promising predictor between TNBC and non-TNBC (AUC = 0.804) or normal samples (AUC = 0.991), respectively (Figure 5D). Of further note, the TNBC-specific CRC TFs also participated in the regulation of gene expression in different biological processes (Figure 5E). For instance, they were both able to regulate RNA splicing (GO:0000377 and GO:0008380); MYBL2 and USF1 could participate in cell cycle regulation (hsa04110); USF1 and SOX4 were capable of affecting the Ephrin receptor signaling pathway (GO:0048013 and R−HSA−2682334); USF1 also specifically impacted on the immune processes (R−HSA−1280215) and oxidative phosphorylation (GO:0006119).

### USF1 shows association with TNBC immunophenotypes

**FIGURE 6:**
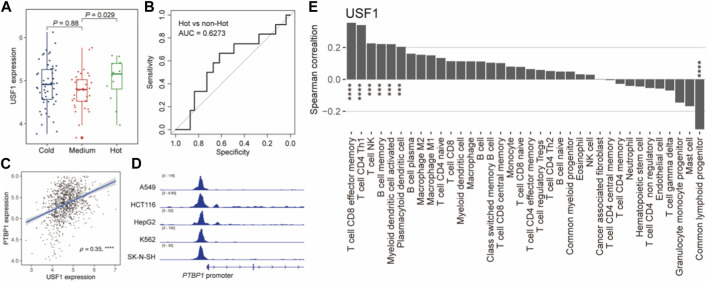
USF1 was associated with TNBC immune microenvironment. **(A)**. Boxplot showing the USF1 expression across TNBC immunophenotypes. *p*-value was calculated by Wilcoxon rank sum test. **(B)**. ROC curves for identifying the immune “Hot” and “non-Hot” samples based on USF1 expression. **(C)**. Scatterplot showing the correlation between USF1 and PTBP1 expression levels. **(D)**. USF1 could bind to PTBP1 promoter. **(E)**. Bar plot showing the Spearman’s correlation between USF1 expression and the infiltration levels. ^*^ *p* < 0.05, ^**^ *p* < 0.001, and ^***^ *p* < 0.0001, as calculated by the Mann–Whitney U test.

We next explored the tumor immunological association of USF1. We first interrogated the immunophenotypes in TNBC samples based on the consensus clustering analysis using the immune cell abundance estimated by xCell ^38^ (Supplementary Figure S6A). A total of three immunophenotypes were identified and their immunoactivity was characterized as “Hot”, “Medium”, and “Cold” (Supplementary Figure S6B). We also observed the expression of USF1 was higher in “Hot” than that in “Cold” (Figure 6A) and exhibitied the potential for differentiating immune “Hot” than other immunophenotypes (AUC = 0.6273) (Figure 6B). Notably, we also found a USF1-downstream gene *PTBP1*, which had been linked to the immune evasion of tumor cell in TNBC (Aran et al., 2017; Orozco et al., 2018). ChIP-seq data showed that USF1 could bind to *PTBP1* promoter regions and potentially involved in modulating *PTBP1* expression (Figures 6C, D). *PTBP1* also showed a significant correlation with tumor purity of TNBC measured by ESTIMATE (Yoshihara et al., 2013) (Supplementary Figure S7A). Moreover, we found both USF1 and PTBP1 expression were significantly associated with the infiltration levels of CD4^+^ Th1 cells that could function in pro-tumor immunity (Lee et al., 2022) (Figure 6E; Supplementary Figure S7B). We subsequently also investigated the prognostic role of downstream genes of core TNBC-TFs supported by ChIP-seq data (ENCODE Project Consortium, 2004) and experiments (Han et al., 2018) in TCGA and METABRIC (Curtis et al., 2012) cohorts of TNBC patients. Several downstream genes of USF1 could act as the prognostic markers, suggesting an overriding function of USF1 in regulating TNBC survival (Supplementary Figure S8). These results suggested that USF1 was an immunophenotype-related CRC TF, and regulated patient’s prognosis in TNBC.

## Discussion

Although the basic mechanisms behind gene transcription are well established (Gifford et al., 2013; Ziller et al., 2015), most cells lack knowledge of the regulatory patterns of the gene expression programs that a few number of transcription factors (TFs) control. Finding CRCs in tumors can provide information on the cellular origin and gene regulatory factors that contribute to the oncogenic state, which may lead to the development of new anti-cancer treatments.

Firstly, the correlation between activated super-enhancers (SEs) and DNA hypomethylation, as well as copy number amplification, suggests a potential link between the abnormal activation of SEs and the dysregulation of key genes and pathways involved in breast cancer development. Genome-wide hypomethylation has been suggested to be an important phenomenon in cancer cells (Nishiyama and Nakanishi, 2021). Notably, a recent study has also reported that enhancer hypomethylation play a pivotal role in driving aberrant transcriptional reprogramming in cancer (Pan et al., 2022). This implies that the aberrant activation of SEs might play a role in the pathogenesis of breast cancer by disrupting normal gene regulation mechanisms. SEs have been shown to play a crucial role in enhancing gene expression (Deng et al., 2020), and their association with DNA hypomethylation and copy number alterations further supports their importance in driving oncogenic processes.

Furthermore, the identification of distinct CRC TFs associated with different breast cancer molecular subtypes provides valuable insights into the heterogeneity of breast cancer. These TNBC-specific CRC TFs may contribute to the specific gene expression programs and regulatory networks underlying TNBC pathogenesis.

Additionally, the role of CRC TFs in immune regulation and immunophenotypes of TNBC was explored. The analysis revealed an association between USF1, a CRC TF, and immune activity in TNBC. USF1 expression was higher in TNBC samples with a “Hot” immunophenotype, characterized by higher immune cell infiltration, compared to samples with a “Cold” immunophenotype. This suggests that USF1 and potentially other CRC TFs may contribute to immune modulation in TNBC, which has implications for understanding the tumor microenvironment and potential immunotherapeutic strategies in TNBC.

Overall, the findings of this study highlight the significance of SEs and CRC TFs in breast cancer, particularly in TNBC. These findings deepen our understanding of the regulatory networks and functional implications of SEs and CRC TFs in breast cancer pathogenesis. Targeting these regulatory elements and their downstream targets could offer novel therapeutic avenues for personalized treatment strategies in breast cancer, especially in TNBC. Further research is warranted to validate the clinical relevance and therapeutic potential of these findings.

## Data Availability

The original contributions presented in the study are included in the article/Supplementary Material, further inquiries can be directed to the corresponding author.
