# Supplementary figures and images for: Super enhancer-driven core transcriptional regulatory circuitry crosstalk with cancer plasticity and patient mortality in triple-negative breast cancer
Source: Front Genet. 2023 Oct 12;14:1258862. doi: 10.3389/fgene.2023.1258862 (PMC10602724; doi:10.3389/fgene.2023.1258862)

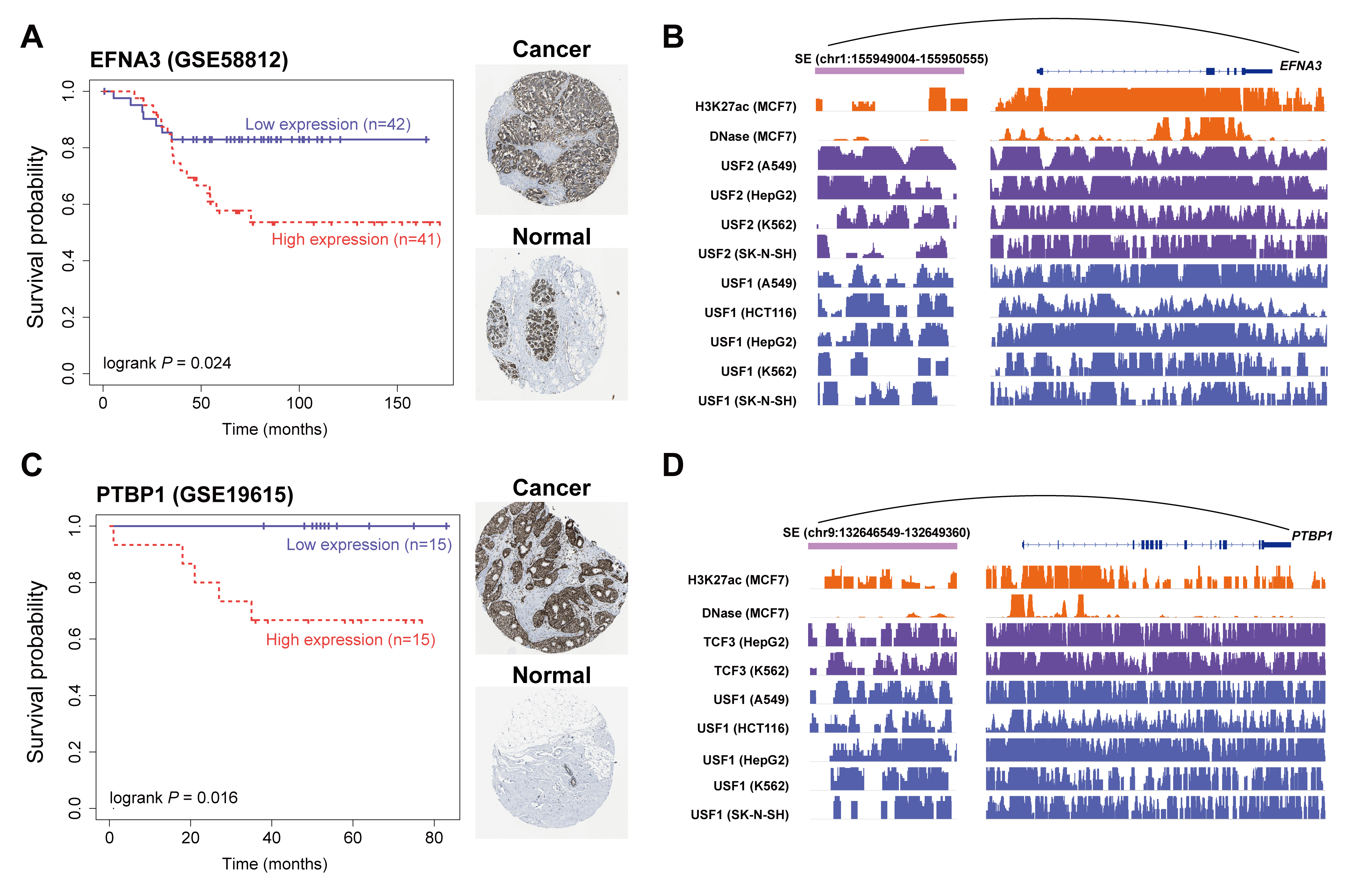

Supplement: Supplementary file 1 [file Image6.tif]

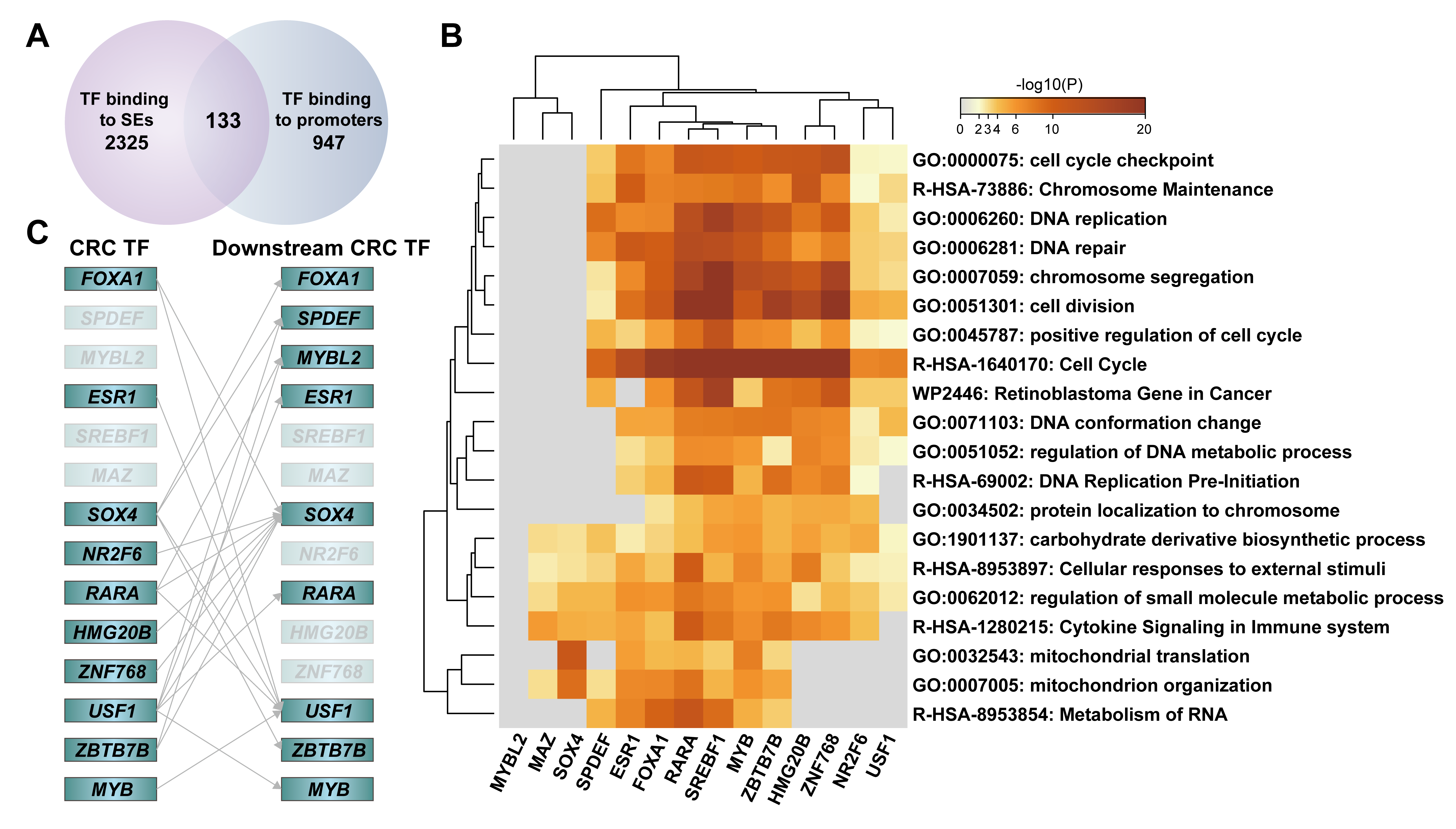

Supplement: Supplementary file 2 [file Image3.TIF]

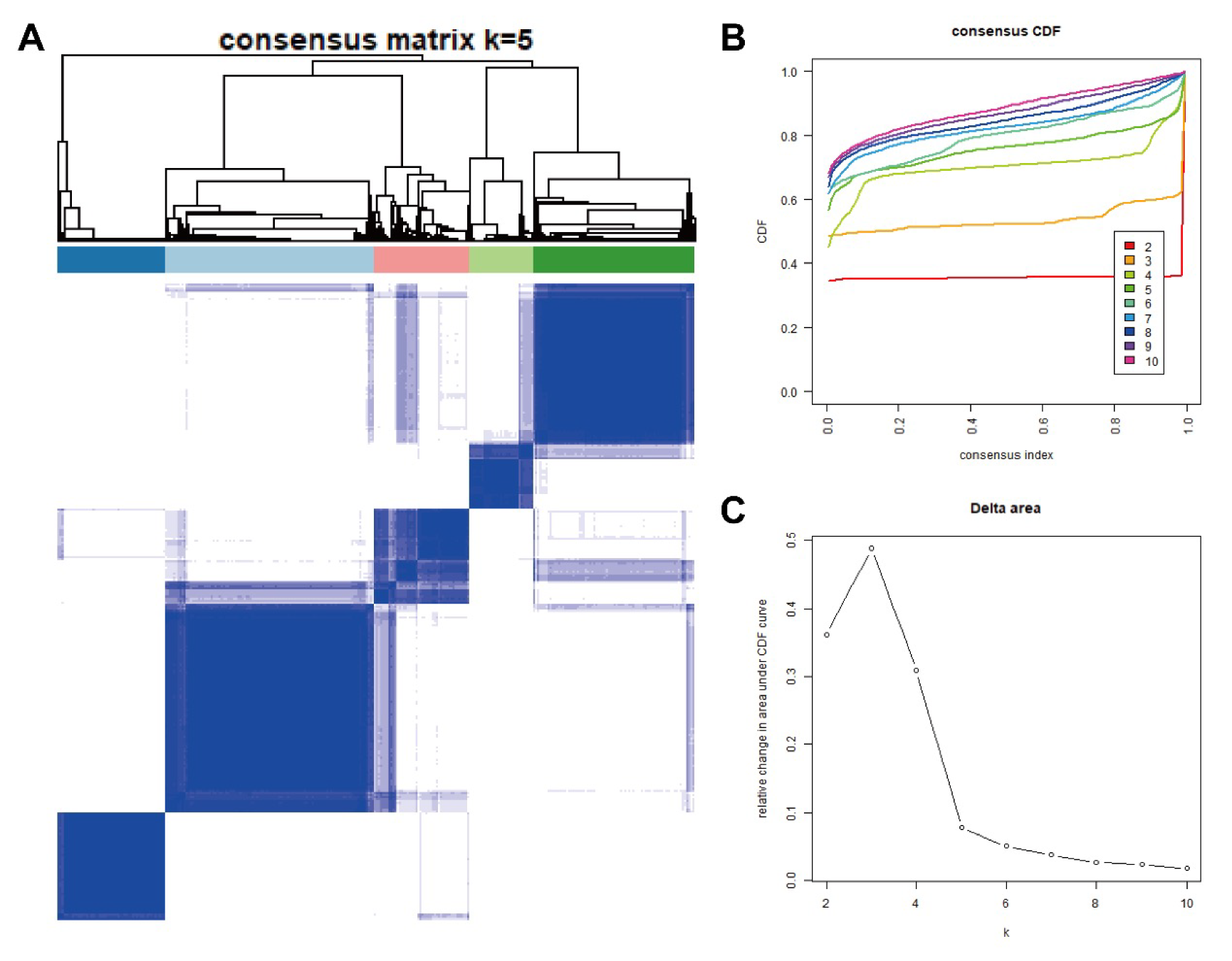

Supplement: Supplementary file 3 [file Image4.tif]

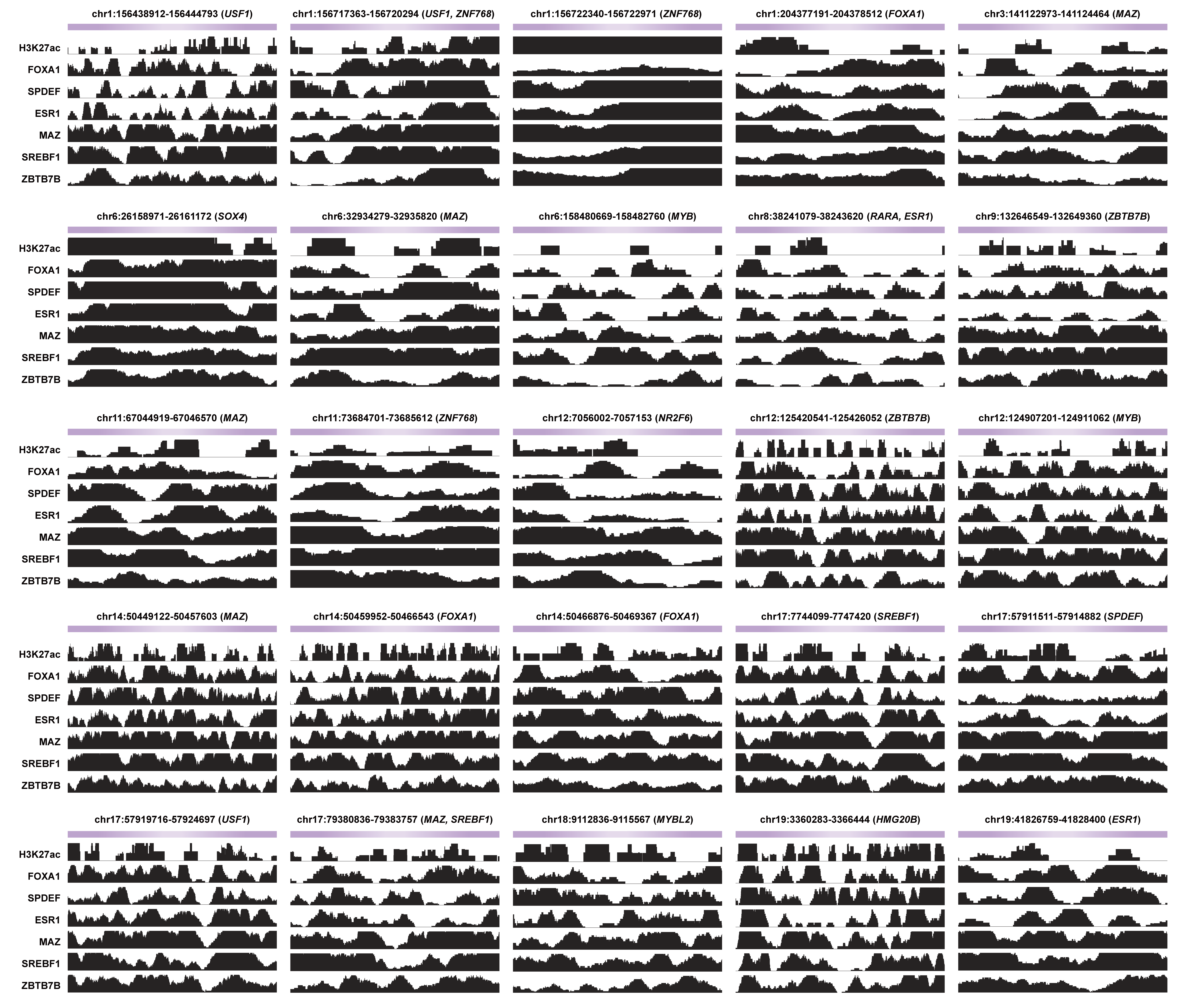

Supplement: Supplementary file 4 [file Image2.TIF]

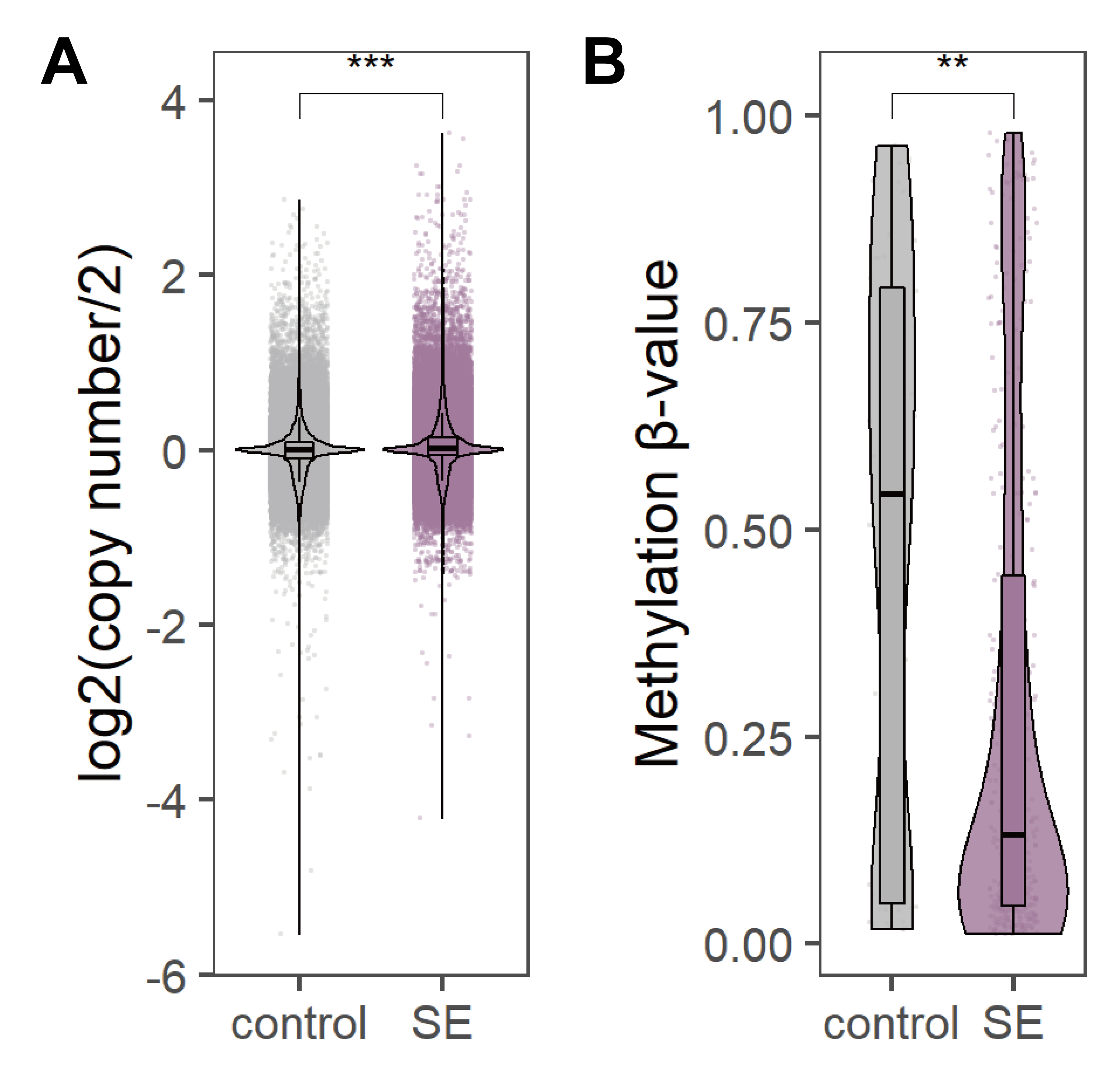

Supplement: Supplementary file 5 [file Image1.TIF]

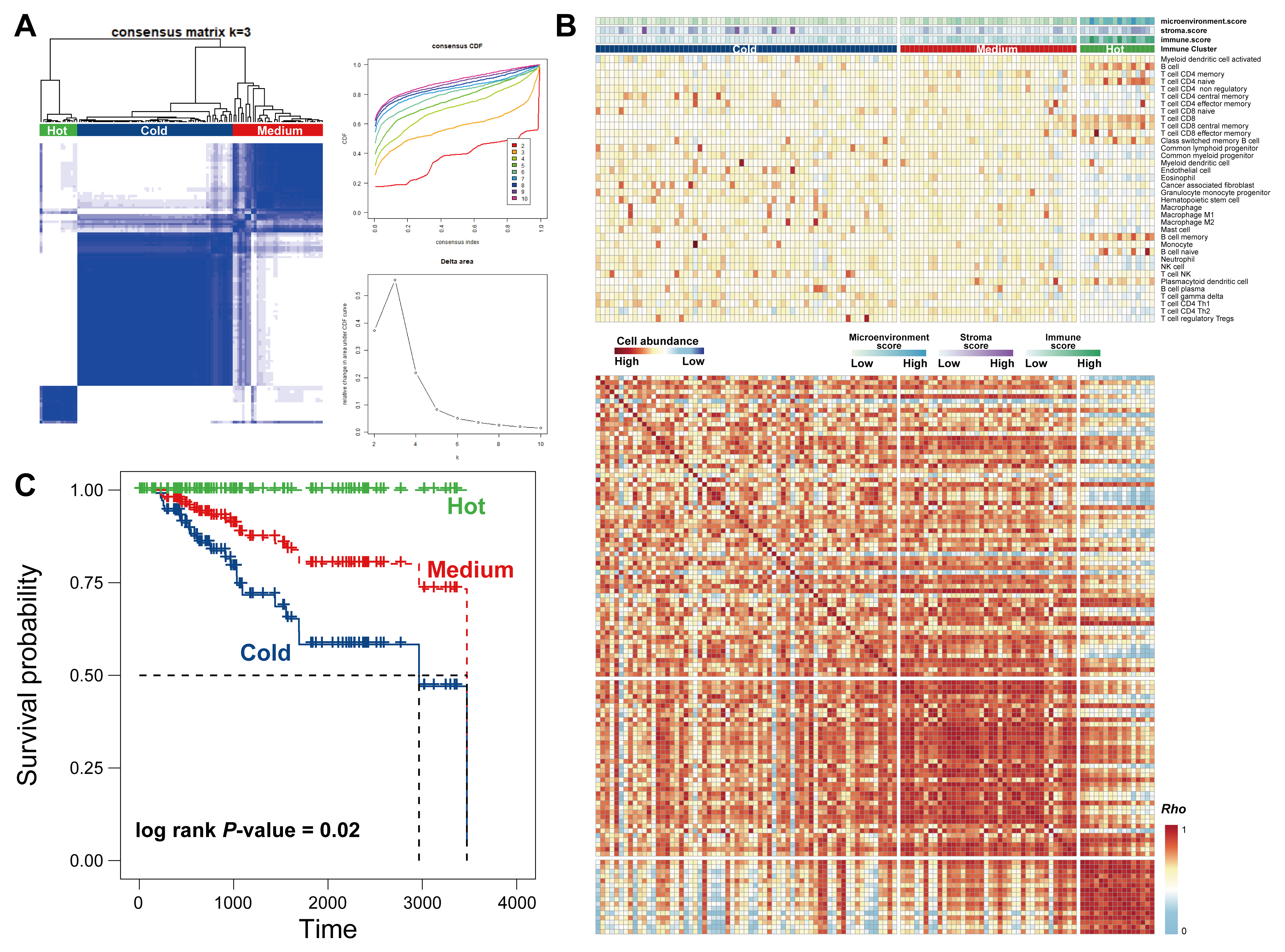

Supplement: Supplementary file 6 [file Image7.tif]

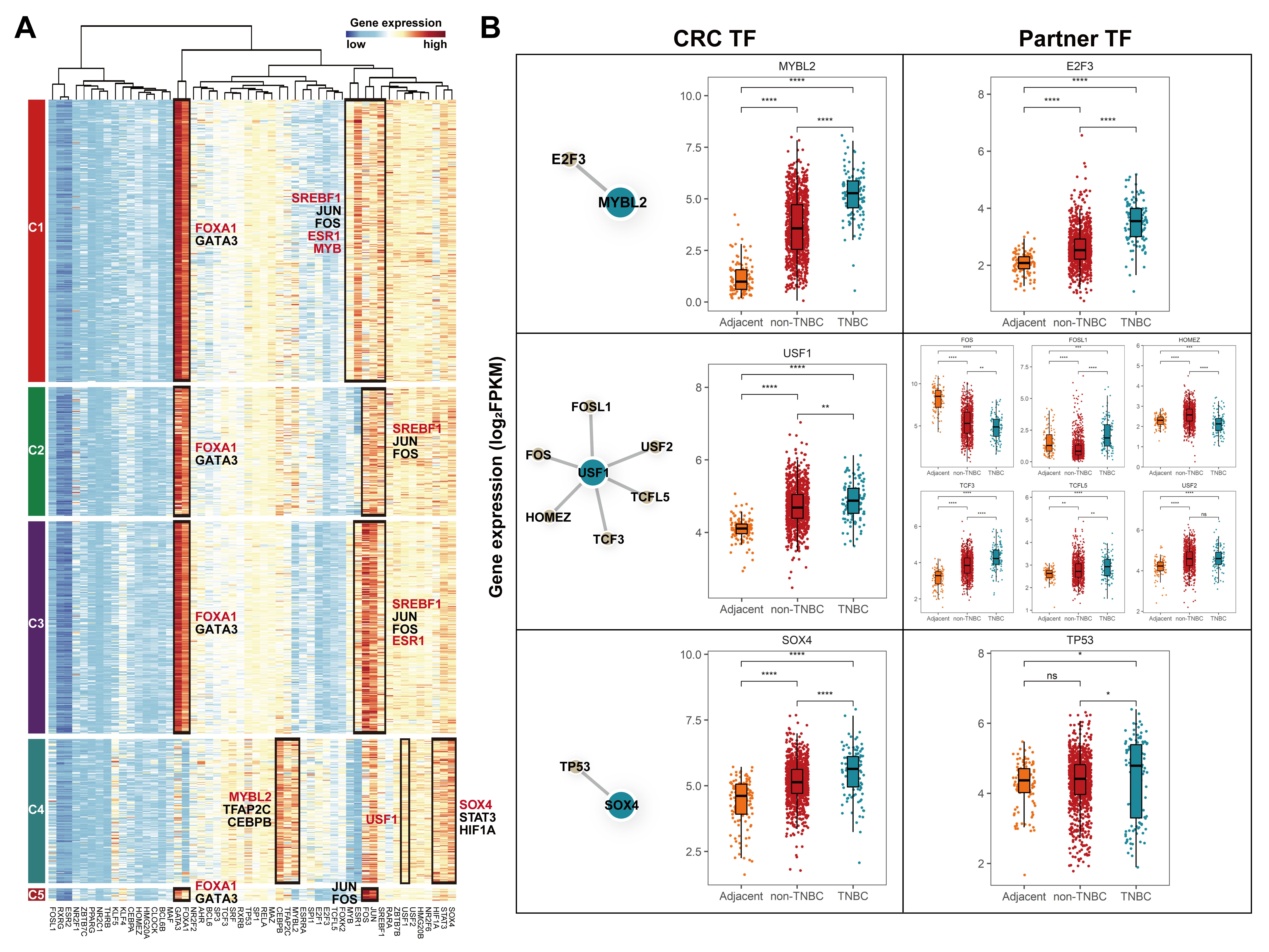

Supplement: Supplementary file 8 [file Image5.tif]
