# Supplementary material for: Super enhancer-driven core transcriptional regulatory circuitry crosstalk with cancer plasticity and patient mortality in triple-negative breast cancer
Source: Front Genet. 2023 Oct 12;14:1258862. doi: 10.3389/fgene.2023.1258862 (PMC10602724; doi:10.3389/fgene.2023.1258862)
Supplement: Supplementary file 9 [file DataSheet1.docx]

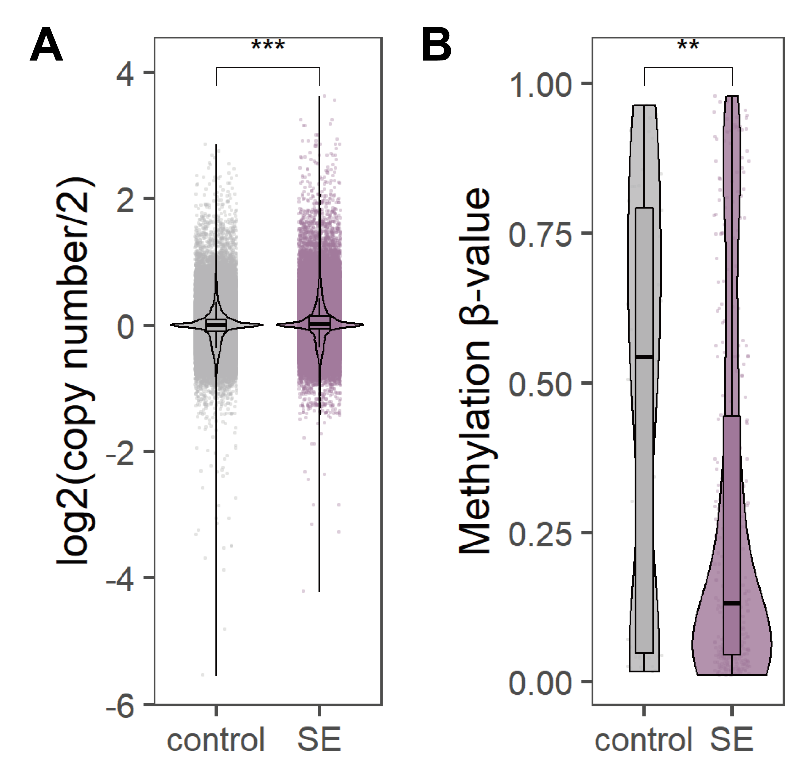


**Figure S1. The copy number variant and DNA methylation levels in SE and random genome regions. A-B.** Boxplot showing the log2 ratio of copy number (A) and methylation β-value (B) in SE (purple) and random genome regions (gray). ^*^*P* < 0.05, ^**^*P* < 0.001, and ^***^*P* < 0.0001, as calculated by the Mann-Whitney U test (A, B).


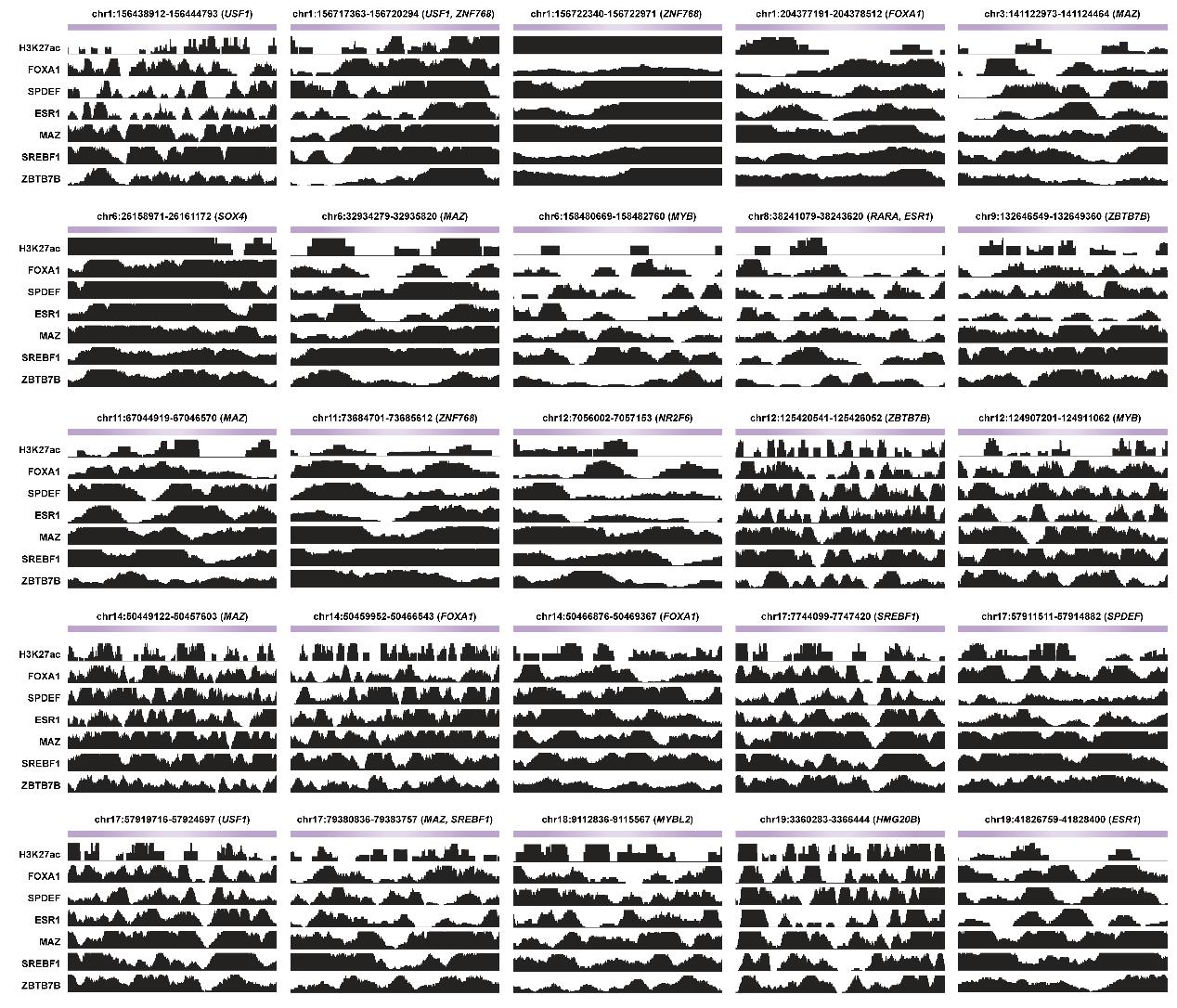


Figure S2. ChIP-seq peaks of H3K27ac (ENCSR017EUK), FOXA1 (ENCSR126YEB), SPDEF (ENCSR042GSX), ESR1 (ENCSR463GOT), MAZ (ENCSR288IJC), SREBF1 (ENCSR197DJH), and ZBTB7B (ENCSR277BXW) to each of the SEs of the CRC TFs in the MCF7 cell lines using ENCODE bigWig files. SE genomic locations are labeled on top of the tracks. The SE-assigned TFs are indicated in brackets.


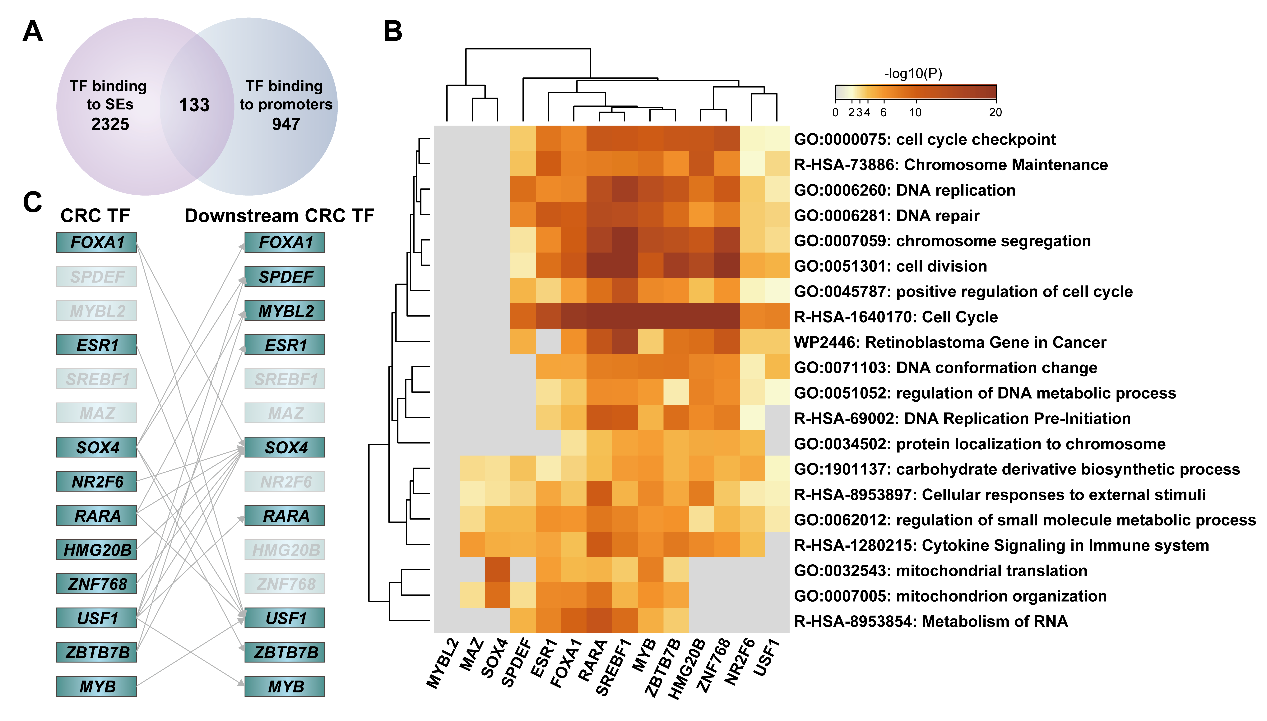


Figure S3. Downstream gene is perturbed by CRC. A. Venn plot showing the number of TF binding locations of downstream genes. B. Heatmap showing the biological functions of each CRC TF could be involved in. C. Schematic diagram of the inter-regulation of CRC TFs.


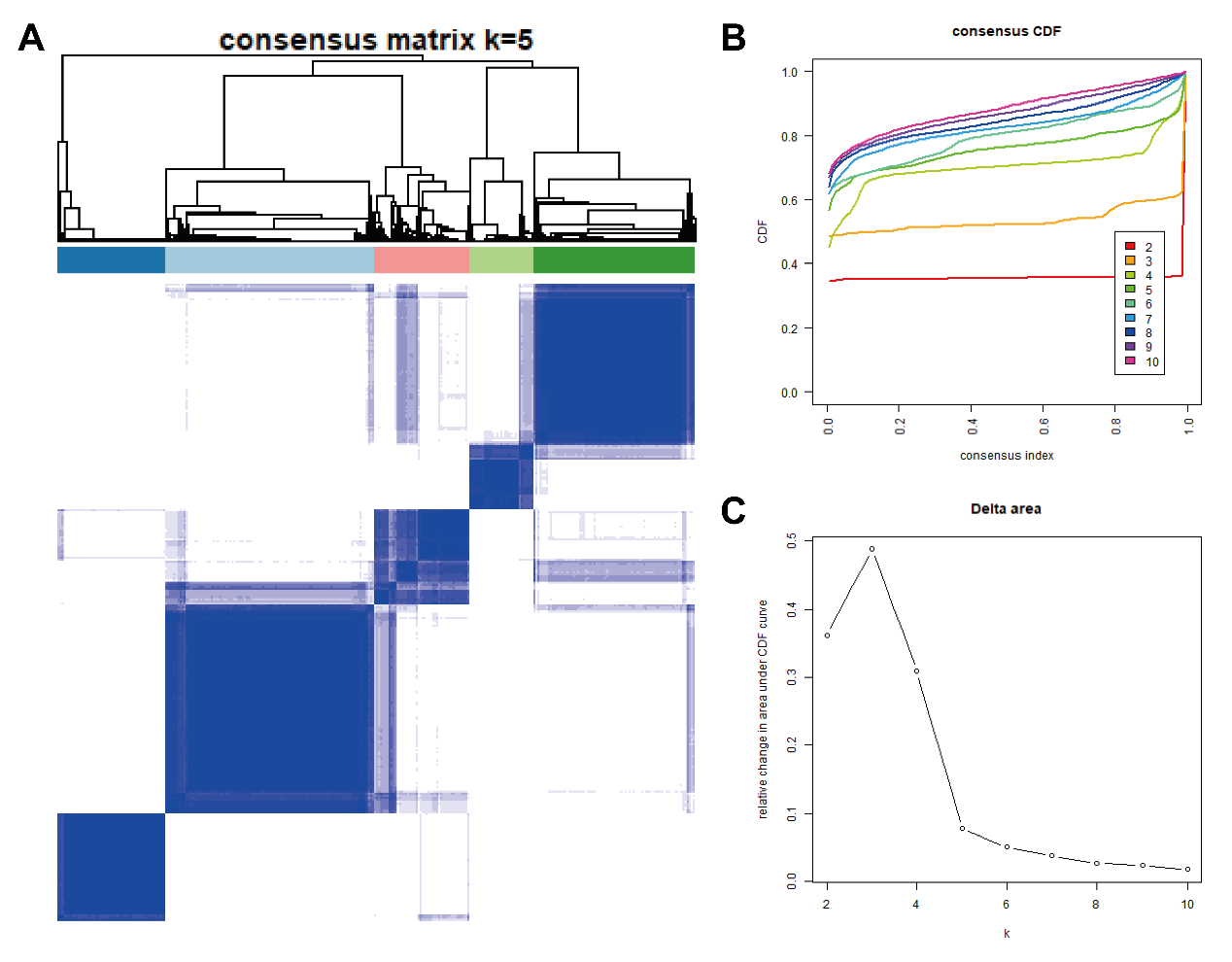


Figure S4. Consensus clustering of cancer samples based on the expression levels of CRC TFs and their partner TFs. A. Heatmap of consensus scores of cancer samples when k = 5 (k was set to 5 because the area under the cumulative distribution function (CDF) curve increased only slightly when k > 5). B. Relative change in area under the CDF curve when the number of clusters (k) equals 2 to 10. C. CDFs of consensus clustering results (k = 2, ..., 10) of cancer samples.


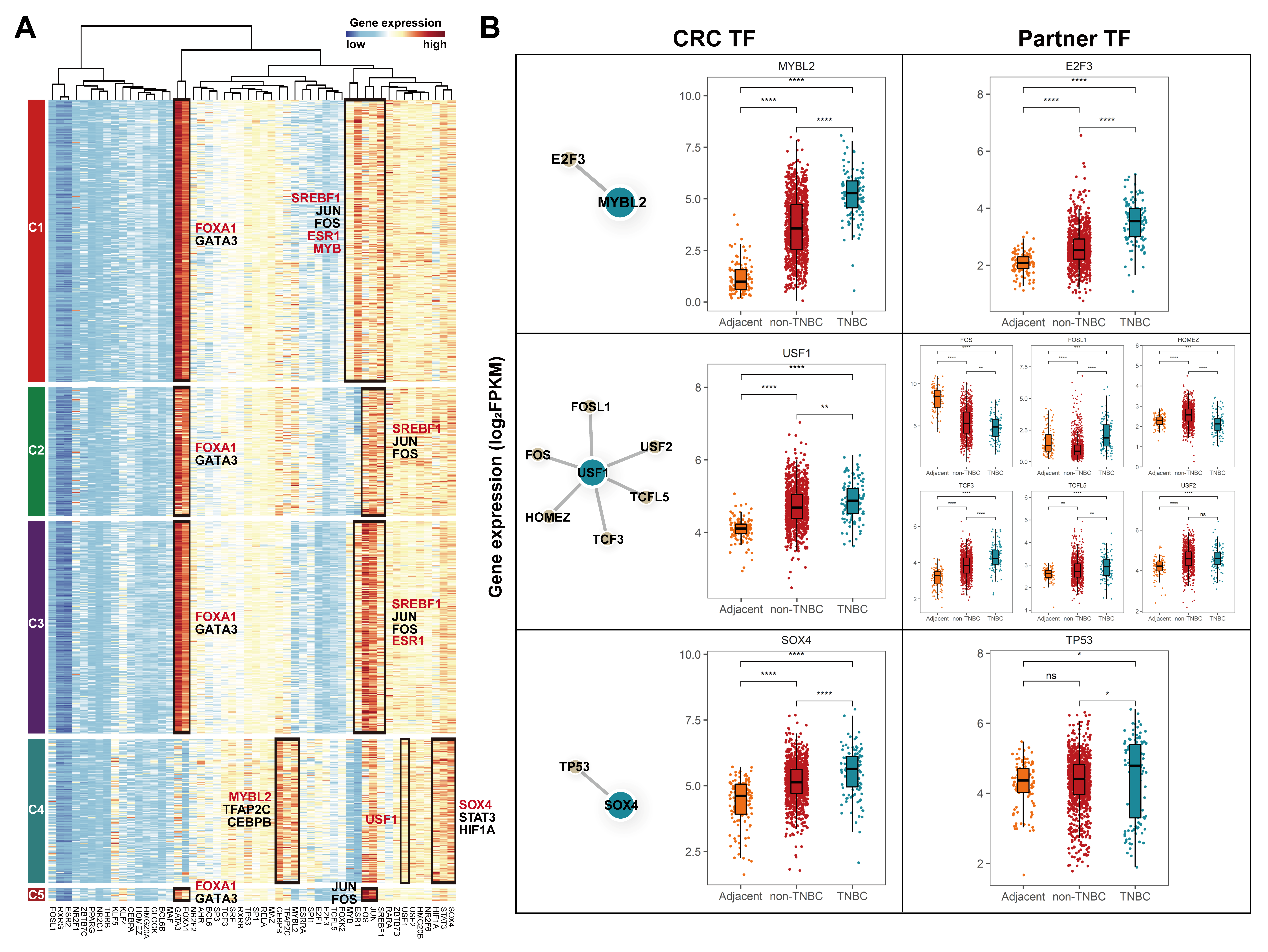


Figure S5. Identification of CRC subtype-specific TFs. A. Heatmap showing the expression levels of CRC TFs and their partner TFs. The subtype-specific TFs were labeled, in which the specific CRC TFs were labeled in red. B. The C4-specific CRC TFs and partner TFs expression levels in TNBC, non-TNBC, and normal samples.


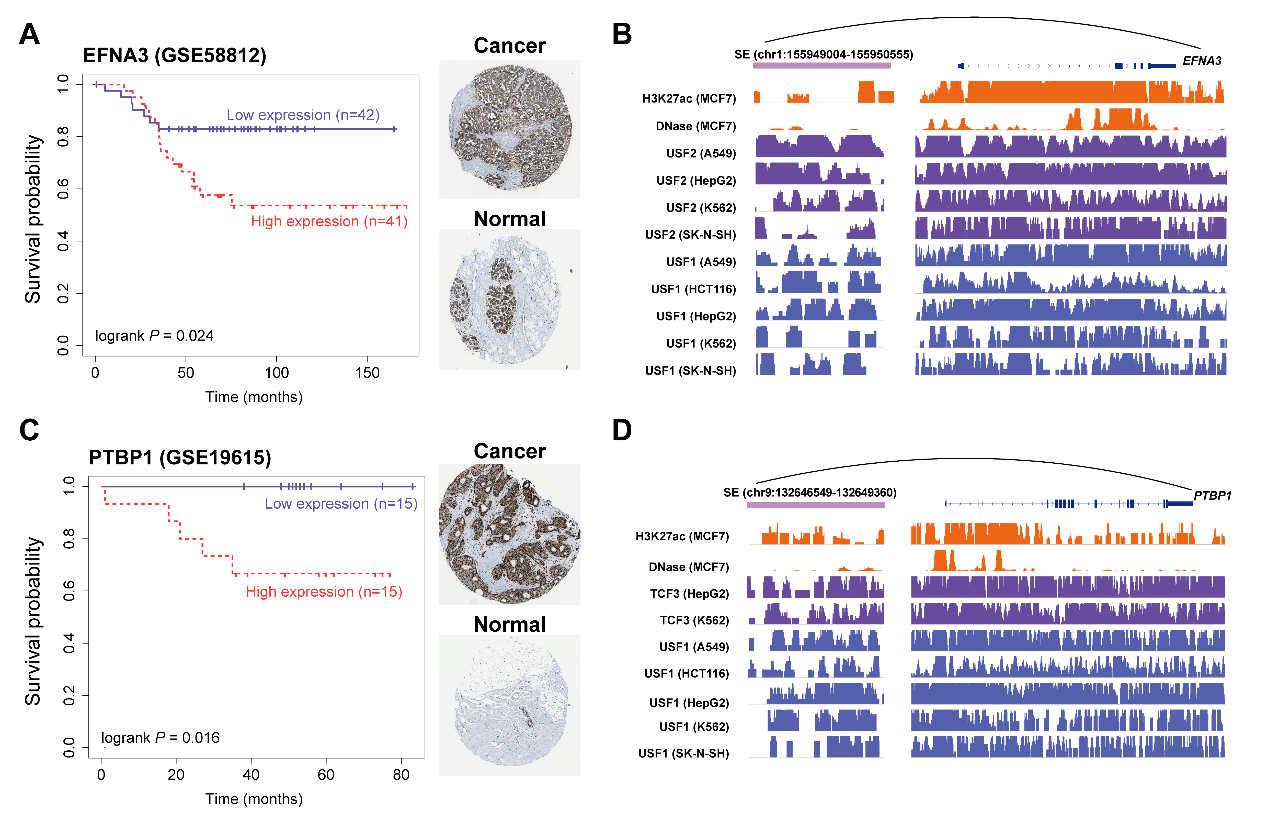


Figure S6. *EFNA3* and *PTBP1* could be regulated by USF1. A. Kaplan-Meier curve for patient stratification based on *EFNA3* expression in an independent TNBC dataset (GSE58812). The IHC staining of EFNA3 protein in both breast cancer samples and normal tissues was shown left. For full IHC protein profiles, view the gene at [www.proteinatlas.org/pathology](http://www.proteinatlas.org/pathology). B. The ChIP-seq peaks of H3K27ac, DNase, USF2, and USF1 in SE and EFNA3 genome regions in cancer cell lines. C. Kaplan-Meier curve for patient stratification based on *PTBP1* expression in an independent TNBC dataset (GSE19615). The IHC staining of PTBP1 protein in both breast cancer samples and normal tissues was shown left. D. The ChIP-seq peaks of H3K27ac, DNase, TCF3, and USF1 in SE and EFNA3 genome regions in cancer cell lines.


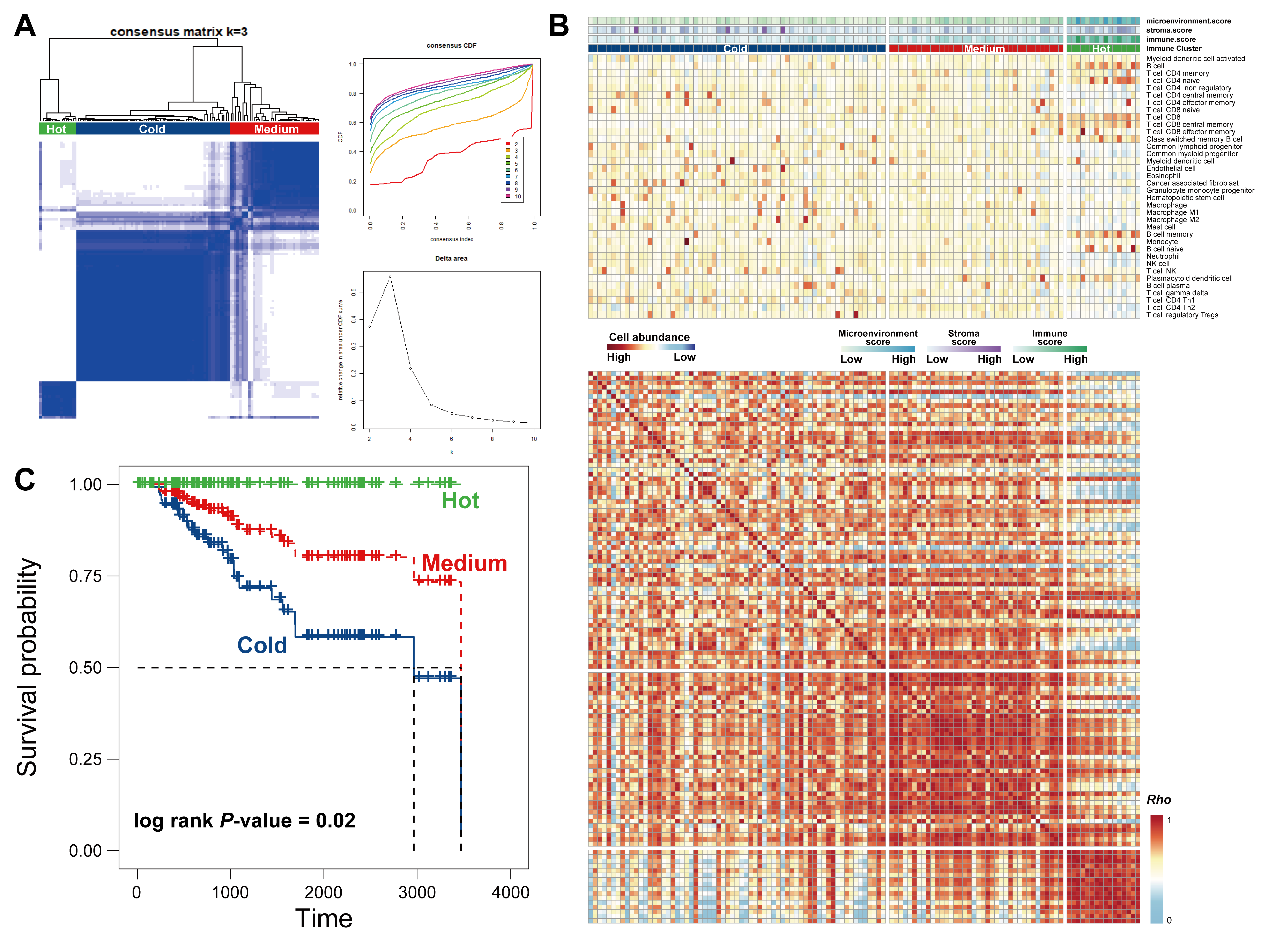


Figure S7. Characterization of the immunophenotypes in TNBC. A. The consensus clustering analysis results are based on the immune cell abundance estimated by xCell. k was set to 3 because the area under the cumulative distribution function (CDF) curve increased only slightly when k > 3). B. Heatmap showing the immune cell abundance (top) and the correlations between TNBC samples. C. The survival curves showing the survival time among the “Hot”, “Medium”, and “Cold” immunophenotypes.


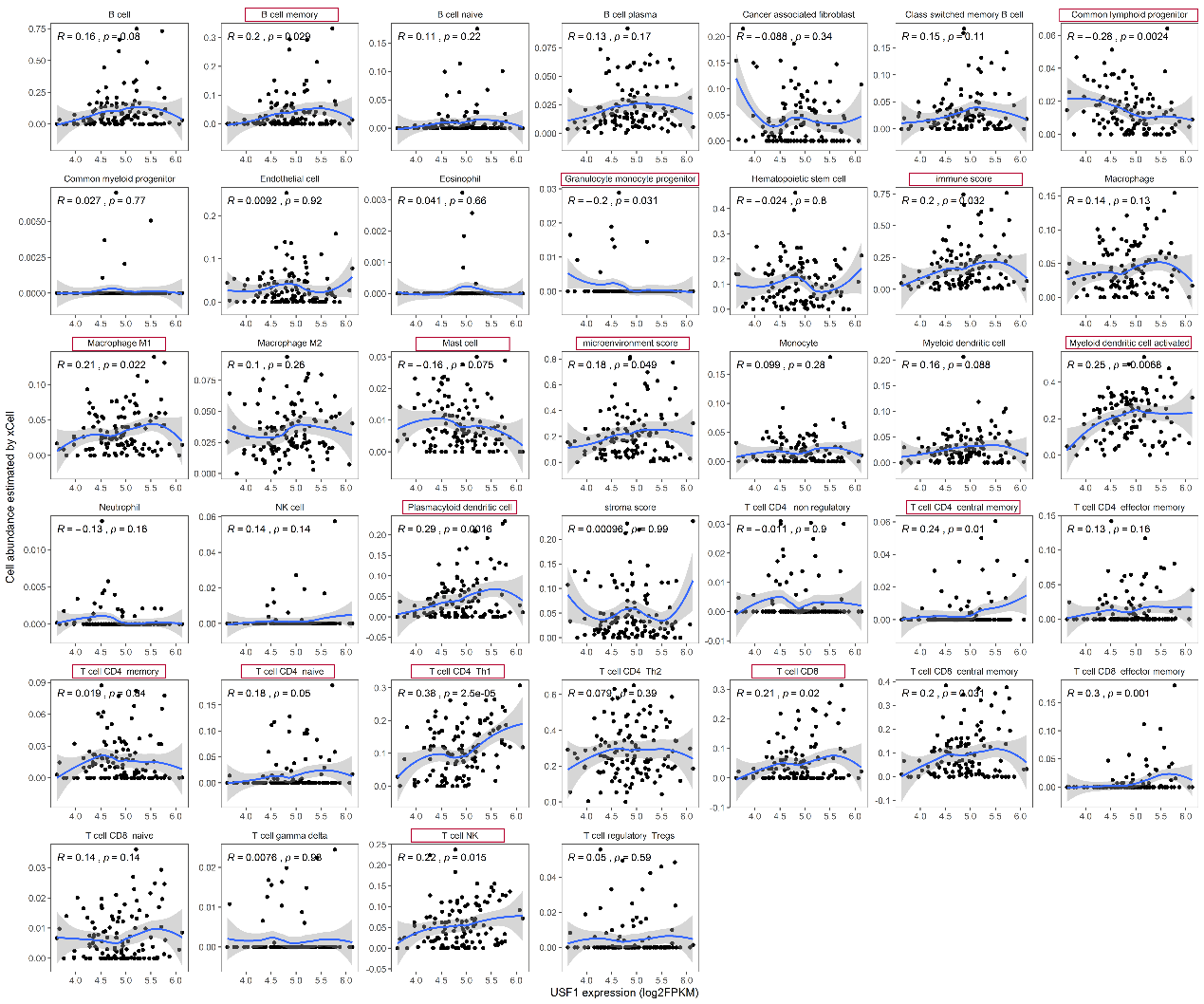


Figure S8. The Spearman’s correlation analysis between USF1 expression and immune cells’ abundance estimated by xCell. Significant associations are boxed in red.
